# The effects of first-line pharmacological treatments for reproductive outcomes in infertile women with PCOS: a systematic review and network meta-analysis

**DOI:** 10.1186/s12958-023-01075-9

**Published:** 2023-03-03

**Authors:** Ge Peng, Zhe Yan, Yuqi Liu, Juan Li, Jinfang Ma, Nanwei Tong, Yan Wang

**Affiliations:** 1 Department of Endocrinology and Metabolism, West China Hospital, Sichuan University, Chengdu, 610041 China; 2 Center for Diabetes and Metabolism Research, West China Hospital, Sichuan University, Chengdu, 610041 China; 3 Department of Gynaecology and Obstetrics, West China 2nd University Hospital, Sichuan University, Chengdu, 610041 China

**Keywords:** Polycystic ovarian syndrome, Infertility, Pharmacological treatment, Network meta-analysis

## Abstract

**Background:**

Polycystic ovarian syndrome (PCOS) is one of the most common causes of infertility in reproductive-age women. However, the efficacy and optimal therapeutic strategy for reproductive outcomes are still under debate. We conducted a systematic review and network meta-analysis to compare the efficacy of different first-line pharmacological therapies in terms of reproductive outcomes for women with PCOS and infertility.

**Methods:**

A systematic retrieval of databases was conducted, and randomized clinical trials (RCTs) of pharmacological interventions for infertile PCOS women were included. The primary outcomes were clinical pregnancy and live birth, and the secondary outcomes were miscarriage, ectopic pregnancy and multiple pregnancy. A network meta-analysis based on a Bayesian model was performed to compare the effects of the pharmacological strategies.

**Results:**

A total of 27 RCTs with 12 interventions were included, and all therapies tended to increase clinical pregnancy, especially pioglitazone (PIO) (log OR 3.14, 95% CI 1.56 ~ 4.70, moderate confidence), clomiphene citrate (CC) + exenatide (EXE) (2.96, 1.07 ~ 4.82, moderate confidence) and CC + metformin (MET) + PIO (2.82, 0.99 ~ 4.60, moderate confidence). Moreover, CC + MET + PIO (2.8, -0.25 ~ 6.06, very low confidence) could increase live birth most when compared to placebo, even without a significant difference. For secondary outcomes, PIO showed a tendency to increase miscarriage (1.44, -1.69 ~ 5.28, very low confidence). MET (-11.25, -33.7 ~ 0.57, low confidence) and LZ + MET (-10.44, -59.56 ~ 42.11, very low confidence) were beneficial for decreasing ectopic pregnancy. MET (0.07, -4.26 ~ 4.34, low confidence) showed a neutral effect in multiple pregnancy. Subgroup analysis demonstrated no significant difference between these medications and placebo in obese participants.

**Conclusions:**

Most first-line pharmacological treatments were effective in improving clinical pregnancy. CC + MET + PIO should be recommended as the optimal therapeutic strategy to improve pregnancy outcomes. However, none of the above treatments had a beneficial effect on clinical pregnancy in obese PCOS.

**Trial registration:**

CRD42020183541; 05 July 2020

**Supplementary Information:**

The online version contains supplementary material available at 10.1186/s12958-023-01075-9.

## Background

Polycystic ovarian syndrome (PCOS) is a heterogeneous disease characterized by hyperandrogenaemia, metabolic disorders, and ovulation disturbance [1]. As a public health issue, PCOS is the leading cause of infertility and affects 8 ~ 13% of reproductively aged women. It has been reported that androgen excess and insulin resistance combined with hypothalamic-pituitary dysfunction lead to ovarian disturbance, which is a major mechanism in anovulation and infertility [2].

The treatment strategies of PCOS vary, but lifestyle intervention is recommended for all PCOS individuals, especially those complicated with obesity and/or insulin resistance. Since the most common cause of infertility in women with PCOS is ovulatory disturbance, ovulation induction agents are prevalently used as first-line pharmacological interventions for women who wish to conceive. Classical ovulation induction agents, including clomiphene citrate (CC) and letrozole (LZ), are widely used in many countries and have different mechanisms of action. CC is a selective estrogen receptor modulator that stimulates follicle-stimulating hormone (FSH) secretion and promotes ovarian follicle maturation by inhibiting the negative feedback of estradiol on gonadotropins, whereas LZ could increase FSH secretion in the pituitary gland by reducing estrogen levels in the blood [3–5].

In addition to CC and LZ, the well-known hypoglycemic drug metformin (MET) was also considered a first-line drug due to its ability to improve ovulation and clinical pregnancy in women with PCOS [6, 7]. Insulin resistance has been proven to play an important role in infertile PCOS individuals. Based on this, any agents that can increase insulin sensitivity, such as thiazolidinediones (TZDs) and glucagon-like peptide 1 receptor agonist (GLP-1RA), are highly attractive and used as one of the first-line pharmacological treatments for infertile PCOS women. If the above first-line ovulation induction agents fail to result in conception, gonadotrophin and assisted reproductive technology are recommended as the second-line strategy by the guidelines [4].

To date, several clinical trials and meta-analyses have analysed the efficiency of different agents in improving pregnancy outcomes in infertile women with PCOS. However, which strategy is the optimal option for improving pregnancy outcomes is still unclear [8]. To answer this question, we conducted a network meta-analysis and systematic review to comprehensively evaluate the effects of first-line ovulation induction agents and insulin sensitizers in infertile women with PCOS. Fallopian tube lesions, endometriosis or other pathological diseases leading to infertility are beyond the scope of this article.

## Materials and Methods

We performed a systemic review and network meta-analysis of placebo-controlled or head-to-head randomized controlled trials (RCTs) according to the PRISMA guidelines [9], which was registered with the PROSPERO international prospective register of systematic reviews (registration number CRD42020183541).

### Search strategy and selection criteria

We searched electronic databases of PubMed, EMBASE, and Web of Science via specific strategies using the keywords “treatment”, “therapy”, “intervention”, “polycystic ovary syndrome” and “randomized controlled trial” to Nov 2021. The detailed search strategy have been listed in Supplementary Table 1.

The included articles met the following criteria: 1) The studies were placebo-controlled or head-to-head RCTs enrolling women who were infertile due to PCOS and did not have diabetes, hyperprolactinemia, thyroid disorders, late-onset congenital adrenal hyperplasia, or Cushing’s syndrome. 2) The studies included at least one of the following interventions: CC, LZ, MET, TZDs (pioglitazone (PIO) or rosiglitazone (ROS)), GLP1-RA. 3) The studies must report the clinical pregnancy number in each group, but not the pregnancy rate per cycle. Clinical pregnancy must have been identified with ultrasound detection of fetal heartbeats.

Studies with the following conditions were excluded: 1) trials that used human chorionic gonadotropin (hCG) or other gonadotropins since they are not first-line treatments. 2) Studies that conducted surgical treatments. 3) Studies in which different medicines were given consecutively according to the individual’s response to the drugs, resulting in different treatments among the study groups. 4) Non-English articles or those without the full text available were not included.

Two reviewers independently screened the titles, abstracts and full texts. Disagreements were resolved by consultation with a third reviewer.

### Outcomes

The primary outcomes were clinical pregnancy confirmed by ultrasound and live birth. Secondary outcomes included miscarriage, ectopic pregnancy, and multiple pregnancy.

### Quality assessment and risk of bias

The quality of the RCTs was assessed independently by two researchers in a blinded fashion with the Cochrane Collaboration tool for assessing the risk of bias in RCTs, which evaluated RCTs from six aspects: adequate random sequence generation, allocation concealment, blinding of participants and personals, blinding of outcome assessment, incomplete outcome data addressed, selective reporting, and other bias [10]. All included studies were allocated into three levels of risk of bias: high, middle, and low risk. We defined high-risk studies as having more than one high-risk mark and low-risk studies as having no high-risk marks and no more than three unclear marks. The remaining studies that were between low and high risk were defined as middle-risk studies. Disagreements were resolved by consultation with a third reviewer. The chart of risk bias assessment was made by RevMan 5.

To assess the certainty of the evidence across RCTs, we applied the Confidence in Network Meta-Analysis (CINeMA) approach with six domains, including within-study bias, reporting bias, indirectness, imprecision, heterogeneity and incoherence, which was conducted in the CINeMA web application and graded the confidence as high, moderate, low, and very low [11]. Publication bias was investigated by the trim-and-fill method and contour-enhanced funnel plots [12].

### Data analysis

Based on the assumptions of homogeneity, similarity and consistency, we conducted a network meta-analysis combining direct and indirect comparisons in a Bayesian model using the R package GeMTC (R package version 0.8–4) [13, 14] with a random-effects model [15]. The effect sizes were log odds ratios (ORs) with 95% credible intervals (CIs) for dichotomous outcomes. If statistically significant, which means interval of 95% CI did not contain zero, the number-needed-to-treat (NNT) or the number-needed-to-harm (NNH) were calculated based on the estimated effect size and the pooled control event rate [16]. A random-effects model was computed using Markov chain Monte Carlo methods with Gibbs sampling based on simulations of 200 00 iterations in each of 4 chains.

We used node splitting to evaluate the homogeneity and consistency and characterized the extent of heterogeneity as low, moderate, and high according to the first and third quantiles of their empirical distributions. Pairwise comparisons were made using a league table expressed as the log OR with 95% CI. A frequency table was constructed from these rankings and normalized by the number of iterations giving the rank probabilities. Convergence was assessed using standard diagnostics [17].

We explored residual heterogeneity by subgroup analysis, and sensitivity analysis was conducted to evaluate the robustness of the model. We defined high heterogeneity as *I*^*2*^ > 50%, middle heterogeneity as 30 ≤ *I*^*2*^ < 50, and low heterogeneity as *I*^*2*^ < 30 [18]. All analyses were repeated in the sensitivity analyses to take into consideration the networks by using different analysis models.

### Meta-regression and subgroup analysis

Meta-regression analyses were performed in in Stata 16 with Metareg packages to explore whether baseline demographic or other factors affect outcomes. To further analyse the heterogeneity, we performed a subgroup analysis according to body mass index (BMI) since obesity is common in PCOS patients. Considering the distinction between East Asia and non-East Asia, we used different BMI cut-off levels to analyse the effect of pharmacological treatments. For East Asia, overweight and obesity were defined as a BMI of 22.5–27.5 kg/m^2^ and a BMI ≥ 27.5 kg/m^2^, respectively [19]. For non-east Asians, those two cut-off points were 25 kg/m^2^ and 30 kg/m^2^, respectively [20].

## Results

### Search results and characteristics of the included studies

**Fig. 1:**
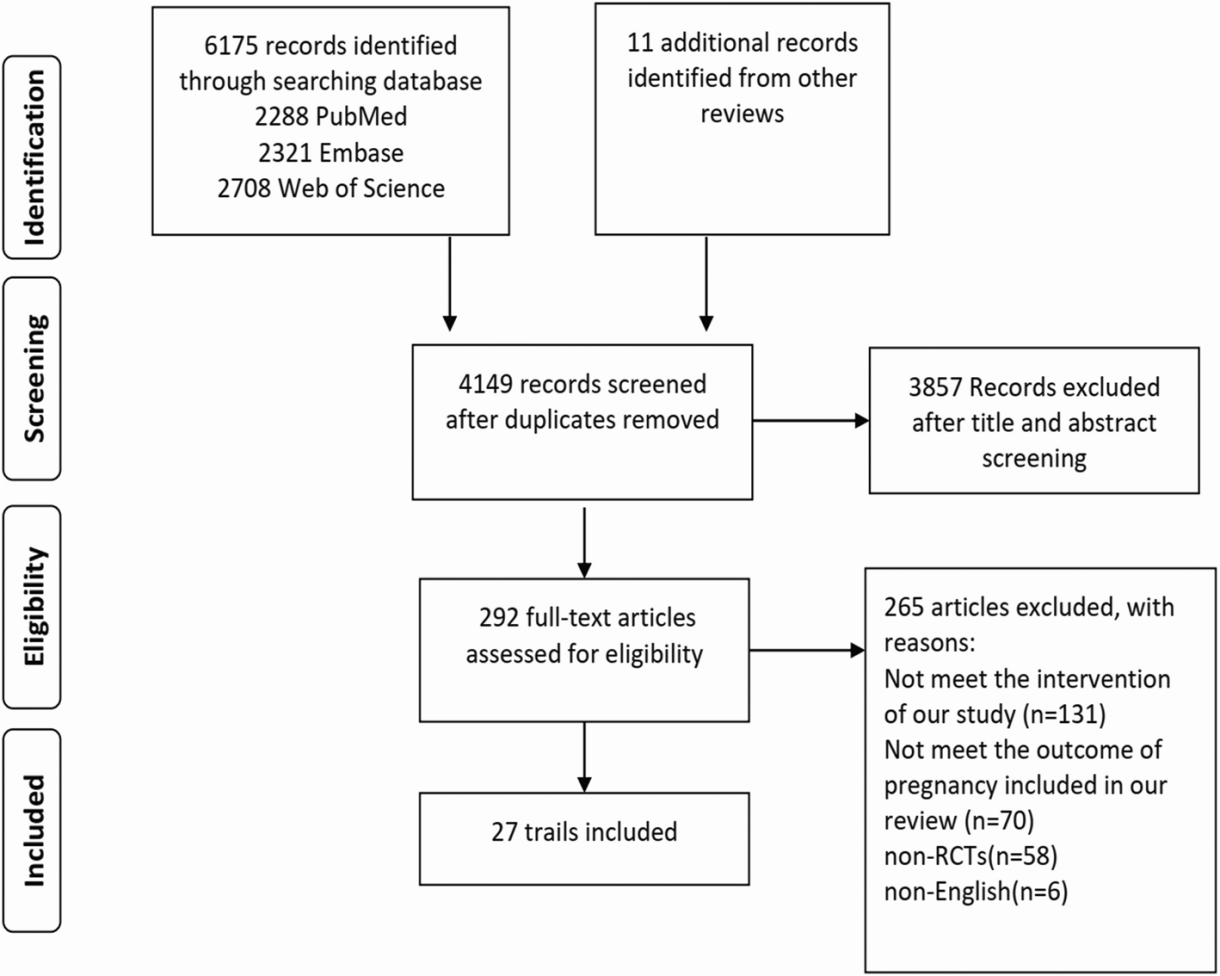
PRISMA flow diagram of screening literatures

The search identified 4149 citations after removing duplicates, among which 27 RCTs [21–47] with 4608 participants met the eligibility criteria and were included in the meta-analysis (Fig. 1). The sample size of the included studies ranged from 25 to 750, and the publication years were 2001 to 2021. The mean age of all participants was 27.67 years, and the mean infertility duration was 2.45 years. The mean BMI of subjects in these RCTs ranged from 20.8 to 38.4 (Supplementary Table 2).

**Fig. 2:**
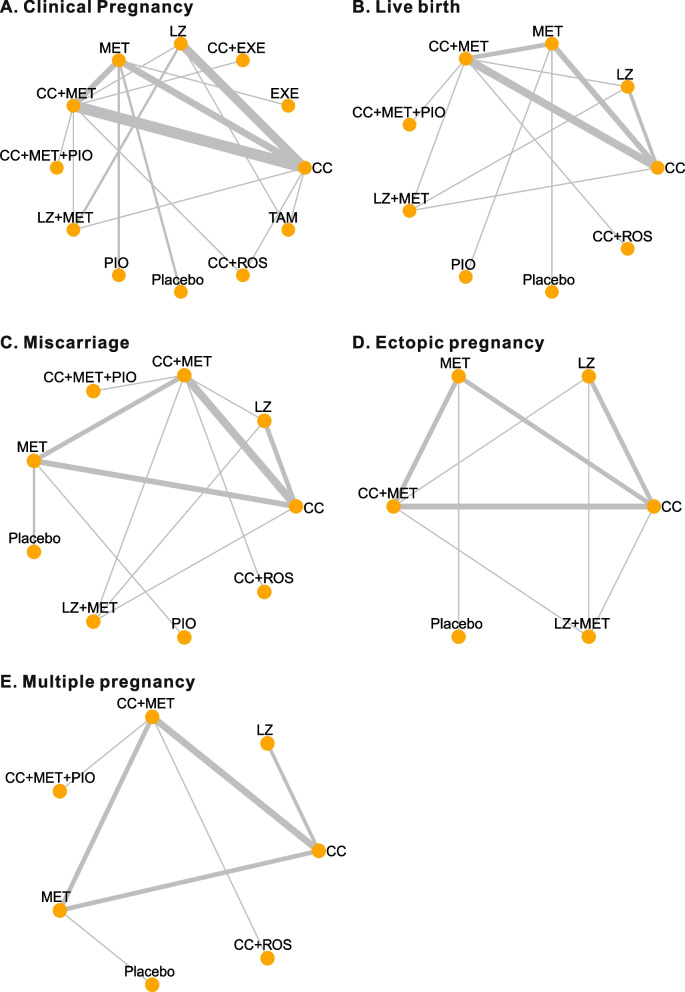
Network plot for the included trials comparing different pharmacological treatments for PCOS women. MET, metformin; CC, clomiphene citrate; PIO, pioglitazone; LZ, letrozole; ROS, rosiglitazone; EXE, exenatide; TAM, tamoxifen

In addition to placebo, a total of 11 types of pharmacological treatments were included in these studies: CC, LZ, MET, PIO, exenatide (EXE), tamoxifen, dual therapy with CC + MET, LZ + MET, CC + ROS, CC + EXE, and triple therapy with CC + MET + PIO. Two placebo-controlled RCTs and 25 head-to-head RCTs were included in our review. CC monotherapy was the most frequently investigated treatment in 18 studies. Dual therapy with CC + MET was involved in 13 studies. LZ and MET were also commonly used: 8 studies used LZ, and 11 studies used MET monotherapy. Five studies used TZDs (PIO or ROS) as monotherapy [39, 43] or in combination with CC [36, 41] or CC + MET [25]. Two studies used EXE as monotherapy or in combination with CC [34, 45]. Only one trial compared tamoxifen (TAM), a synthetic antiestrogen drug, with CC and LZ [38]. The network of all comparisons for the primary and secondary outcomes for efficacy are presented in Fig. 2.

### Effects of first-line pharmacological treatments in clinical pregnancy

**Fig. 3:**
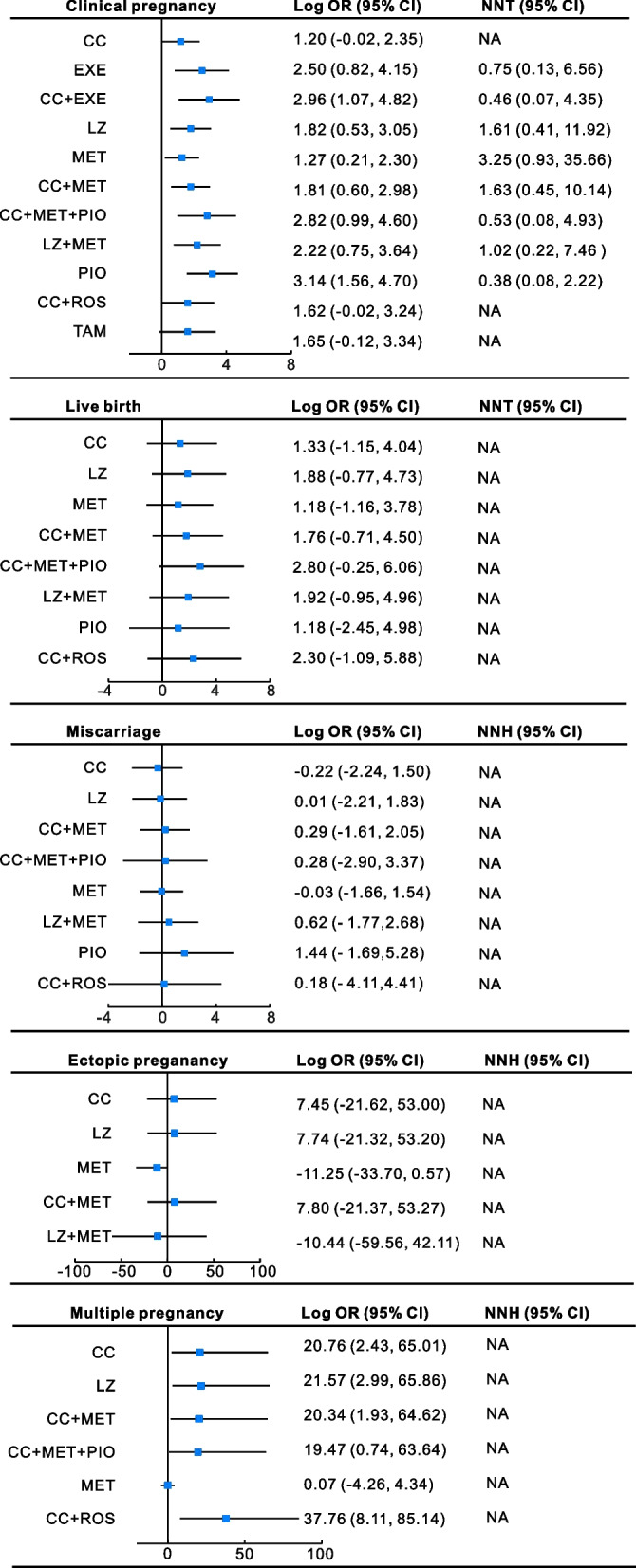
Forest plot for outcomes of different pharmacological treatments compared with placebo. OR, odds ratio; CI, credibility interval; NNT, number needed to treat; NNH, number treated to harm; NA, not applicable

All 27 trials compared each intervention or placebo clinical pregnancy in women with PCOS. Network meta‐analysis showed that most pharmacological treatments (LZ, MET, PIO, EXE, CC + MET, LZ + MET, CC + EXE and CC + MET + PIO) resulted in a significantly elevated clinical pregnancy in infertile women with PCOS compared with placebo (Fig. 3A). The pioglitazone (log OR 3.14, 95% CI 1.56 ~ 4.70; NNT 0.38; moderate confidence), CC + EXE (log OR 2.96, 95% CI 1.07 ~ 4.82, NNT 0.46, moderate confidence) and CC + MET + PIO (log OR 2.82, 95% CI 0.99 ~ 4.60, NNT 0.53, moderate confidence) groups obtained a superior clinical pregnancy than the other interventions. However, there was no statistically significant impact of CC alone (log OR 1.20, 95% CI -0.02 ~ 2.35, very low confidence), CC + ROS (log OR 1.62, 95% CI -0.02 ~ 3.24, very low confidence), or TAM alone (log OR 1.65, 95% CI -0.12 ~ 3.34, very low confidence) on clinical pregnancy (Fig. 3A, Supplementary Table 5A).

**Fig. 4:**
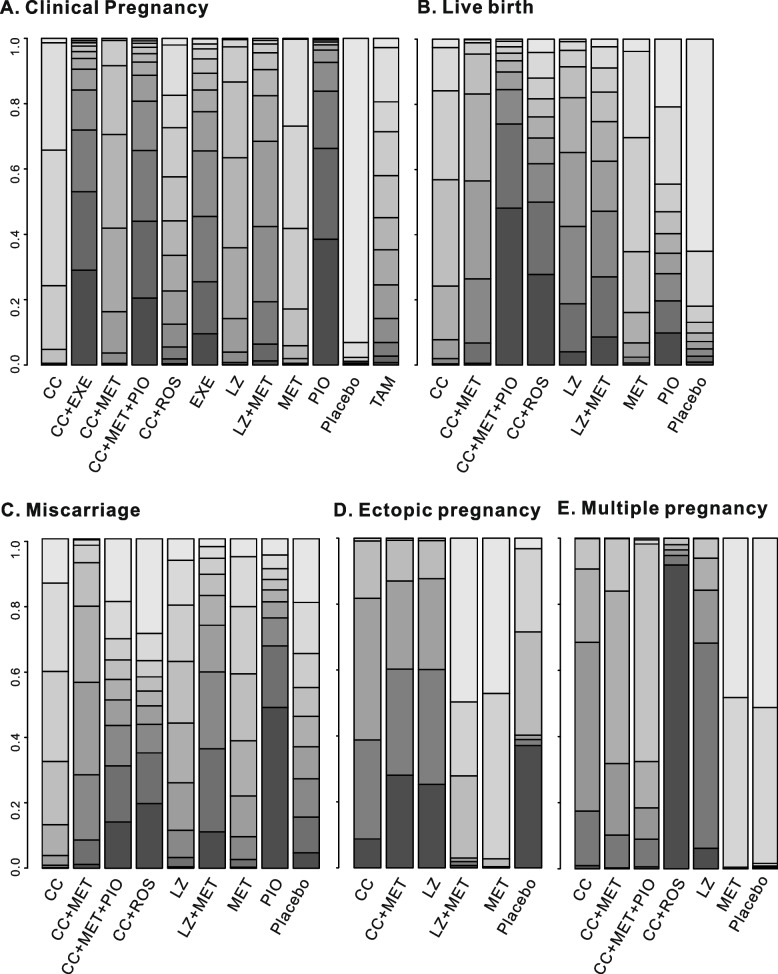
The ranking probability histogram for the outcomes. The color of the bar indicates the possibility rank of outcomes and the larger the proportion of dark colors, the higher the ranking

The league table demonstrated that CC alone tended to be less effective than the other monotherapies or combination treatments, especially CC + EXE, LZ, CC + MET, CC + MET + PIO, LZ + MET, and PIO. However, there was no significant difference in most pairwise comparisons of these pharmacological interventions (Supplementary Table 3A). In addition, the possibility rank suggests that among the 11 pharmacological interventions, pioglitazone, CC + EXE, CC + MET + PIO and EXE might increase the clinical pregnancy most effectively for infertile PCOS women (Fig. 4A).

### Effects of first-line pharmacological treatments on live birth rate

Thirteen studies evaluated the effect of eight pharmacological treatments, including CC, LZ, MET, PIO, CC + MET, LZ + MET, CC + ROS and CC + MET + PIO, on the live birth rate. A trend toward an improved live birth rate in patients who had received pharmacological therapy was noted, but it was not found to be statistically significant (Fig. 3B). It is noted that CC + MET + PIO (log OR 1.62, 95% CI -0.25 ~ 6.06, very low confidence) could increase the live birth rate most when compared to placebo (Supplementary Table 2B, 5B), and the probability rank also suggested that CC + MET + PIO was superior for improving the live birth rate (Fig. 4B). The league table showed that all pairwise comparisons were not statistically significant (Supplementary Table 3B).

### Effects of first-line pharmacological treatments on miscarriage rate, ectopic pregnancy and multiple pregnancy

The miscarriage rate of 8 interventions, which was identical to the live birth rate, was compared in 15 trials. Most of these pharmacological treatments played relatively minor roles in miscarriage when compared with the placebo group (Fig. 3C). However, PCOS patients who received PIO showed a tendency to have an increased miscarriage rate (log OR 0.15, 95% CI -3.64 ~ 4.36, very low confidence), which requires more direct evidence (Fig. 4C, Supplementary Table 3C, 5C).

Only 8 studies provided the outcome of ectopic pregnancy involving five interventions: CC, LZ, MET, CC + MET, and LZ + MET. None of the five interventions increased ectopic pregnancy for PCOS women compared to placebo. Among these, MET (log OR -11.25 95% CI -33.7 ~ 0.57, low confidence) and LZ + MET (log OR -10.44 95% CI -59.56 ~ 42.11, very low confidence) seemed to be beneficial in decreasing ectopic pregnancy (Fig. 3D).

For multiple pregnancy, six interventions, including CC, LZ, CC + MET, CC + MET + PIO, MET, and CC + ROS, were assessed. All of them except MET alone (log OR 0.07 95% CI -4.26 ~ 4.34, low confidence) significantly increased the multiple pregnancy rate (Fig. 3E). However, it made no sense to estimate the effect of ectopic pregnancy and multiple pregnancy in this network meta-analysis because the occurrence of the two adverse events was extremely low in these studies.

### Heterogeneity, inconsistency, and sensitivity assessment

The inconsistency test showed that the clinical pregnancy and live birth rate results were consistent in the direct, indirect and network analyses except for the comparison of CC + MET and MET (Supplementary Fig. 1), and the sensitivity analysis, which used frequentist methods to perform the network meta-analysis, showed similar results, even though the 95% CI was narrower than the results from the Bayes method (Supplementary Fig. 2).

However, significantly high heterogeneity existed in the comparison of CC and MET and MET and CC + MET in the primary outcome. Clinical pregnancy also had high heterogeneity in the comparison between MET and placebo. The heterogeneity in the comparison of CC with CC + MET was moderate, and the other comparisons had low heterogeneity in the analysis (Supplementary Fig. 3A&B). Nevertheless, the heterogeneity was decreased to some extent after subgroup analysis (Supplementary Fig. 3F&G).

For the secondary outcome, heterogeneity was low to moderate for most trials (Supplementary Fig. 3C-E). Unfortunately, sensitivity assessment and subgroup analysis were not accomplished due to the lack of data for the second outcome.

### Meta-regression and subgroup analysis

The results of the meta-regression showed that baseline demographic characteristics, including age, BMI, region, duration of infertility and length of treatment, had no effect on clinical pregnancy and live birth (Supplementary Table 3). However, the potential effect of the variables could not be fully excluded based on negative results because meta-regression may underestimate some small associations. Thus, we performed subgroup analysis with different BMI cut-off points for pregnancy outcome.

The results showed that only one East Asian study included a population with normal BMI, while one East Asian and eight non-East Asian trials included obese populations. In addition, one East Asian study and twelve non-East Asian studies included overweight patients. To obtain sufficient data for the network meta-analysis, we finally divided these studies into obese and nonobese subgroups. In the obese group, no significant difference was found among these medications, including CC, EXE, LZ, MET, CC + MET, PIO, and CC + ROS, when compared with placebo (Supplementary Fig. 4A), whereas in the nonobese group, these medications showed an effect of increasing the clinical pregnancy in women with PCOS except for CC and TAM (Supplementary Fig. 4B).

### Quality assessment and the risk of bias

Only seven studies were at a low risk of bias for all components and seven studies were at a high risk of bias. The main biases were caused by failure of blinding of the participants and personnel and incomplete outcome data. For blinding of the outcome assessment, due to the particularity of pregnancy, we considered all studies to have a low risk of bias in this aspect (Supplementary Fig. 5).

The funnel plot and test were visually symmetrical, which indicated that there was no publication bias in the primary outcome (Supplementary Fig. 6). The CINeMA confidence of results showed moderate to very low confidence grades, mainly due to concerns about within-study bias, heterogeneity and imprecision owing to low numbers of trials for some comparisons (Supplementary Table 5).

## Discussion

Anovulatory infertility is the most prevalent and severe complication of women with PCOS, and approximately 70% of patients with PCOS are infertile [48]. Multiple medications, including classic ovulation induction agents and insulin sensitizers, have been used to improve the pregnancy outcome for those patients as first-line treatment strategies. However, there is insufficient evidence to define an optimal therapy strategy due to few RCTs comparing all interventions head-to-head. Therefore, we systematically evaluated the effects of all first-line pharmacological treatments on reproductive outcomes in PCOS individuals by conducting a network meta‐analysis that can make indirect comparisons among different treatments. To the best of our knowledge, this is the first network meta-analysis to compare the effect of first-line pharmacological agents on pregnancy outcomes in infertile PCOS women. We included clinical pregnancy and live birth as primary endpoints. Miscarriage, ectopic pregnancy, and multiple pregnancy were also assessed as secondary outcomes.

Through the analysis, we found that PIO showed an absolute advantage in improving clinical pregnancy. Pioglitazone is a highly selective synthetic agonist of the nuclear transcription factor peroxisome proliferation-activated receptor gamma (PPAR-γ), which is widely used as an insulin sensitizer in type 2 diabetes. Insulin resistance is a key risk factor involved in the menstrual cycle and reproductive health in PCOS women. Insulin sensitizers can reduce the circulating insulin level, which may play a beneficial role in anovulatory infertility. Previous studies found that PIO ameliorated menstrual cycle abnormalities and triggered ovulation better than metformin in patients with PCOS [49], which is consistent with our results. Our recent study demonstrated that improving insulin sensitivity was superior to weight loss in increasing the clinical pregnancy of obese or overweight PCOS women [50], which might explain the phenomenon of PIO alone presenting a superior effect in clinical pregnancy than other pharmacological interventions. However, we found that PIO alone showed a relative weakness in live birth and a higher miscarriage rate than other interventions. Considering that both clinical pregnancy and live birth are critical endpoints of pregnancy outcomes, PIO alone was not recommended as the optimal choice for infertile PCOS women. The additional reason for the limited use of PIO is medication safety. Currently, TZDs are still listed as pregnancy category C drugs by the Food and Drug Administration (FDA) [51] due to concerns of severe adverse effects.

For the live birth rate endpoint, the combined intervention CC + MET + PIO showed a relative superiority in the eight treatment strategies. It is also presented with an encouraging improvement in clinical pregnancy and a neutral effect on miscarriage rate compared with placebo. Therefore, this triple-combination intervention should be recommended as the optimal therapeutic strategy.

Another medicine worth noting is GLP-1RA – exenatide. Although GLP-1RA was only approved for use in type 2 diabetes and obesity patients, some studies have suggested GLP-1RA to be a promising medicine for women with PCOS because of its excellent weight loss effect and improvement of insulin resistance, which may benefit pregnancy outcomes [52, 53]. A recent systematic review recommended GLP-1RA as a preferable choice over metformin for obese PCOS patients, especially for those with severe insulin resistance [54]. In this study, we also compared the efficiency of EXE in terms of reproductive outcomes. We found that both CC + EXE and EXE alone showed a significant improvement in clinical pregnancy in PCOS women, which was superior to either MET alone or CC + MET. In particular, the CC + EXE group was next only to PIO alone. However, no data presented the effect of GLP-1RA alone or combined therapy in live birth, miscarriage or ectopic pregnancy in those patients, which reduced the quality of the evidence for the recommendation of this drug. As an anti-diabetic medicine, EXE has not been approved to use for fertility treatment in PCOS women in the present, further studies for exploring the effectiveness and safety of EXE in infertile women with PCOS were needed.

We also tried to determine whether the effects were influenced by BMI, and subgroup analysis indicated that pharmacological treatments showed better effectiveness for nonobese patients. It was difficult to improve the clinical pregnancy in obese PCOS patients (BMI ≥ 27.5 for East Asian subjects and BMI ≥ 30 kg/m^2^ for non-East Asian subjects). Other studies have also found that PCOS patients who did not respond to ovulation treatments such as CC were likely to be obese [55]. There is an inverse relationship between increasing BMI and fertility, and the degree of insulin resistance or other metabolic disorders is more severe in obese women [56], which may partly explain the drug-resistant anovulation in PCOS women with obesity.

There are still some limitations in this analysis. First, some arms had few direct comparison data, which caused inconsistencies between direct and indirect results. We finally chose the direct results as the more credible in these cases, such as the live birth in the comparison of metformin and a combination of CC and metformin. Second, although we used strict inclusion criteria, heterogeneity also existed in some arms, which may be due to differences in baseline characteristics. We also failed to quantitively analyse some factors that may affect outcomes such as dosages and metabolic baseline characteristics. Fortunately, the heterogeneity was mitigated to some extent after subgroup analysis with BMI and region. Third, due to a lack of blinding, few trials were assessed to have a low risk of bias, which affected the confidence of the results. Additionally, for feasibility reasons, our work focused on the primary outcomes, but the data for secondary outcomes based on smaller sample sizes were too small to perform sensitivity and subgroup analyses, which may contribute to more bias. Finally, our conclusions between active drugs are mostly based on indirect comparisons, for example, the comparison between PIO and other drugs or placebo, highlighting the need for future clinically relevant and head‐to‐head trials.

## Conclusions

In conclusion, first-line pharmacological treatments have beneficial effects in improving clinical pregnancy for PCOS patients with infertility based on moderate to very low confidence evidence. Moreover, a combination of insulin sensitizers and conventional ovulation stimulants is more effective than monotherapy. The triple-combination treatment “CC + MET + PIO” presented the most stable advantages in both clinical pregnancy and live birth and should be recommended as the optimal first-line therapeutic strategy for infertile PCOS women. GLP-1RA also presented an encouraging effect in clinical pregnancy, however, few data on live birth and miscarriage outcomes limited the usage of this kind of medication. In addition, the subgroup analysis showed that none of the above treatments had a beneficial effect on clinical pregnancy in obese PCOS, which might be the consequence of severe insulin resistance and other metabolic disorders. More studies are required to explore the definite mechanism.

## Supplementary Information

**Additional file 1: Supplementary Table 1. **Search strategies. **Supplementary Table 2.** The characteristics of the included studies. **Supplementary Table 3.** League table of outcomes. **Supplementary Figure 1.** The consistency test of different interventions. **Supplementary Figure 2.** The forest of pregnancy rate using frequentist method. **Supplementary Figure 3.** The heterogeneity test of different interventions. **Supplementary Table 4.** Network meta-regression analysis of outcomes. **Supplementary Figure 4.** Forest plot for Subgroup analysis of pregnancy. **Supplementary Figure 5.** Risk of bias assessment in the RCT. **Supplementary Figure 6.** The adjusted-funnel plot of primary outcome. **Supplementary Table 5.** Confidence in effect estimates.

## Data Availability

All data is available in this paper.

## Abbreviations

PCOS: Polycystic ovarian syndrome
BMI: Body mass index
CC: Clomiphene citrate
CI: Confidence intervals
EXE: Exenatide
FDA: The Food and Drug Administration
GLP-1RA: Glucagon-like peptide 1 receptor agonist
LZ: Letrozole
MET: Metformin
NNH: Number-needed-to-harm
NNT: Number-needed-to-treat
ORs: Odds ratios
PIO: Pioglitazone
PPAR-γ: Peroxisome proliferation-activated receptor gamma
RCTs: Randomized controlled trials
ROS: Rosiglitazone
TAM: Tamoxifen
TZDs: Thiazolidinediones
FSH: Follicle-stimulating hormone
CINeMA: Confidence in Network Meta-Analysis
