# Supplementary material for: The effects of first-line pharmacological treatments for reproductive outcomes in infertile women with PCOS: a systematic review and network meta-analysis
Source: Reprod Biol Endocrinol. 2023 Mar 3;21:24. doi: 10.1186/s12958-023-01075-9 (PMC9983155; doi:10.1186/s12958-023-01075-9)
Supplement: Supplementary file 1 — Additional file 1: Supplementary Table 1. Search strategies. Supplementary Table 2. The characteristics of the included studies. Supplementary Table 3. League table of outcomes. Supplementary Figure 1. The consistency test of different interventions. Supplementary Figure 2. The forest of pregnancy rate using frequentist method. Supplementary Figure 3. The heterogeneity test of different interventions. Supplementary Table 4. Network meta-regression analysis of outcomes. Supplementary Figure 4. Forest plot for Subgroup analysis of pregnancy. Supplementary Figure 5. Risk of bias assessment in the RCT. Supplementary Figure 6. The adjusted-funnel plot of primary outcome. Supplementary Table 5. Confidence in effect estimates. [file 12958_2023_1075_MOESM1_ESM.pdf]

Supplementary materials

content

Supplementary Table 1. Search strategies 2
Supplementary Table 2. The characteristics of the included studies 4
Supplementary Table 3. League table of outcomes 10
Supplementary Figure 1. The consistency test of different interventions 15
Supplementary Figure 2. The forest of pregnancy rate using frequentist method 17
Supplementary Figure 3. The heterogeneity test of different interventions 18
Supplementary Table 4. Network meta-regression analysis of outcomes 28
Supplementary Figure 4. Forest plot for Subgroup analysis of pregnancy 29
Supplementary Figure 5. Risk of bias assessment in the RCTs 30
Supplementary Figure 6. The adjusted-funnel plot of primary outcome 31
Supplementary Table 5. Confidence in effect estimates 32

Supplementary Table 1. PICO framework and Search strategies

PICO framework

P = Patient / population, PCOS patients with infertility

I = Intervention, first-line pharmacological interventions

C = Comparison, placebo

O = Outcome, reproductive outcomes

1A. PubMed database search strategy

Number	Search terms
#1	"polycystic ovary syndrome"[MeSH Terms]
#2	"polycystic ovary syndrome"[Title/Abstract]
#3	"PCOS"[Title/Abstract]
#4	“treatment” [Title/Abstract]
#5	“therapy” [Title/Abstract]
#6	‘intervention” [Title/Abstract]
#7	"randomized control"[Title/Abstract]
#8	"randomised control"[Title/Abstract]
#9	"random*" [Title/Abstract]
#10	"RCT" [Title/Abstract]
#11	#1 OR #2 OR #3
#12	#4 OR #5 OR #6
#13	#7 OR #8 OR #9 OR #10
#14	#11 AND #12 AND #13

1B. Embase database search strategy

Number	Search terms
#1	'ovary polycystic disease'/exp
#2	pcos:de,ab,ti OR pcod:de,ab,ti OR pco:de,ab,ti
#3	therapy:de,ab,ti OR treatment:de,ab,ti OR intervention:de,ab,ti
#4	randomized controlled trial'/exp
#5	'controlled clinical trial'/exp
#6	randomized:de,ab,ti OR randomly:de,ab,ti OR trial:ti OR plecebo:de,ab,ti OR rct:de,ab,ti
#7	#1 OR #2
#8	#4 OR #5 OR #6
#9	#3 AND #7 AND #8

1C. Web of science search strategy

Number	Search terms
#1	(TS=(("polycystic ovary syndrome"))) OR TS=(PCOS)
#2	(TS=(("polycystic ovary syndrome"))) OR TS=(PCOS)
#3	((TS=(("randomized control"))) OR TS=(RCT)) OR TS=(random*)
#4	#1 AND #2 AND #3

Supplementary Table 2. The characteristics of the included studies

Study/ Author	No. participants	Longest treatment	Outcomes	Drug	Doses of drugs①	Mean Age (year)	Mean BMI	Duration of infertility (years)
Amer 2017[21]	159	6 cycles	Primary outcome: clinical pregnancy; secondary outcomes: ovulation, live-birth (LB), pregnancy per ovulating patient, pregnancy per strata, mono-ovulation, endometrial development, pregnancy outcome and pregnancy complications	LZ②<br>CC③	2.5-5mg/d<br>50-100mg/d	28.3<br>28.1	27.5<br>27.7	1.5<br>1.5
Ayaz 2013[22]	42	6 cycles	Menstrual Cycle, ovulatory Responses, conception	CC<br>CC+MET	50 -150 mg/d<br>CC, 50 -150 mg/d<br>MET, 1500mg/d continuously	31.5<br>32	NA④<br>NA	NA<br>NA

① Letrozole and clomiphene citrate were used for 5 days in one cycle unless presented separately.

② LZ, letrozole

③ CC, clomiphene citrate

④ NA, not applicable

				⑤				
Bansal 2021[23]	90	3 cycles	Primary outcome: endometrial thicknesses; secondary outcomes: ovulation rate, monofollicular development, pregnancy rate, and time to pregnancy assessment	LZ	2.5-7.5mg/d	27.0	23.9	3.90
				CC	50 -150 mg/d	26.0	23.1	3.40
Fatima 2018[24]	128	3 cycles	Conception rate	CC	50 -150 mg/d	28.67	NA	3.89
				CC+MET	CC, 50 -150 mg/d<br>MET, 1500mg/d continuously	28.55	NA	3.19
Fattah GA[25] 2014	100	3 cycles	Primary outcome: ovulation rate; secondary outcomes: number of follicles, serum E2, endometrial thickness, pregnancy rate, liver and kidney functions	CC+MET	CC,50-150mg/d<br>MET, 1500mg/d continuously	26.9	27.44	3.38
				CC+MET +PIO⑥	CC,50-150mg/d<br>PIO, 15mg/d, 10 days/cycle<br>MET, 850 mg/d, 10 days/cycle	25.8	27.2	2.87
Ghahiri 2016[26]	101	4 cycles	Pregnancy, abortion or ectopic pregnancy	CC	100mg/d	25.63	27.13	NA
				LZ	5mg/d	25.63	28.24	NA
Johnson 2010[27]	171	6 cycles	Primary outcomes: clinical pregnancy, live birth; secondary outcomes: adverse events, ovulation, spontaneous abortion, ectopic pregnancy, multiple pregnancy and other adverse	CC	50-150mg/d	28.2	26.2	NA
				MET	MET,1500mg/d over 2 weeks	28.9	26.5	NA
				CC+MET	CC,50-150mg/d<br>MET, 1000mg/d over 2 weeks	29.2	26.9	NA
				Placebo	NA	29.2	37.6	NA
				MET	MET,1000mg/d over 2 weeks	29.5	38	NA

⑤ MET, metformin

⑥ PIO, pioglitazone

			complications					
Kar 2015[28]	80	6 cycles	Primary outcome: live birth rate; secondary outcomes: ovulation rate, pregnancy rate, and early pregnancy loss rate	CC	50–150 mg/d	25.8	26.5	2.75
				MET	850-1700 mg/d, over 2 weeks	26.62	24.5	1.70
				CC+MET	CC, 50–150 mg/d<br>MET, 850-1700 mg/d, over 2 weeks	25.2	27.2	2.53
Karimzadeh 2007[29]	200	3 cycles	Ovulation rate, pregnancy rate	MET	500-1500/d, continuously	27.2	28.8	5.6
				Placebo	NA	28.6	29.49	6.2
Karimzadeh 2010[30]	268	6 cycles	Pregnancy rates, multiple pregnancy rates	CC	100mg/d	27.47	27.2	4.1
				MET	500-1500/d, continuously	27.33	27.17	3.9
				CC + MET	CC, 100mg/d<br>MET,500-1500/d, continuously	27.34	27.96	4.55
Legro 2007[31]	626	6 cycles	Primary outcome: live births; secondary outcomes: the rate of pregnancy loss, singleton birth, and ovulation	CC	50mg/d	27.9	36	3.45
				MET	500-2000mg/d, continuously	28.1	35.6	3.25
				CC+MET	CC, 50mg/d<br>MET,500-2000mg/d, continuously	28.3	34.2	3.39
Legro 2014[32]	750	5 cycles	The primary outcome: live birth; secondary outcomes: ovulation, pregnancy loss, singleton birth, and congenital anomalies	CC	50 mg/d	28.8	35.1	3.5
				LZ	2.5 mg/d	28.9	35.2	3.4
Liu C 2017[33]	268	3 cycles	Ovulation rate, pregnancy rate, and pregnant outcome (abortion, premature delivery, and live birth)	CC	CC, 50-150 mg/d	26.8	21.1	1
				CC+MET	CC, 50-150 mg/d<br>MET,1000-1500mg/d, continuously	27.2	21.4	1

				LZ	5 mg/d	27	20.8	1
				LZ+MET	LZ, 5 mg/d<br>MET,1000-1500 mg/d, continuously	27.2	21.6	1
Liu, X 2017[34]	176	3 cycles	Weight changes, fat mass changes, metabolic changes, endocrine changes, inflammatory marker, cycle changes, pregnancy rates	MET	MET,500-1000 mg/d, continuously	27.7	28.29	NA
				EXE[7]	10-20 ug/d, 12weeks	27.9	29.16	NA
Mohsen 2012[35]	100	3 cycles	Primary outcome: ovulation; secondary outcomes: pregnancy and changes in fasting serum glucose and fasting insulin.	CC	100 mg/d	26.4	32	1.77
				CC+ROS[8]	CC, 100mg/d<br>ROS,8mg/d, continuously	25.9	31.7	1.85
Mohsin 2019[36]	100	3 cycles	Pregnancy	LZ	5mg/d	28.28	26.01	3.11
				LZ+MET	LZ, 5mg/d<br>MET, 1500mg/d, continuously	28.74	25.49	3.15
Moll 2008[37]	225	6 cycles	Pregnancy rates	CC	50–150 mg/d	28.4	27.8	1.3
				CC+MET	CC, 50–150 mg/d<br>MET, 2000mg/d, continuously	27.9	28.5	1.6
Moussa 2016[38]	150	6 cycles	Primary outcomes: endometrial thickness and endometrial blood flow; secondary outcomes: the development and number of follicles, and the pregnancy rate.	CC	100mg/d	27.5	26.9	NA
				LZ	5mg/d	27.2	26.8	NA
				TAM[9]	40	27.5	26.7	NA

⑦ EXE, exenatide
⑧ ROS, rosiglitazone
⑨ TAM, tamoxifen

Ortega Gonzalez C 2005[39]	25	6 cycles	Metabolic and hormonal parameters, pregnancy occurrence and outcomes	MET<br>PIO	2550 mg/d, continuously<br>30 mg/d, continuously	29<br>28.8	34.1<br>32.2	NA<br>NA
Palomba 2005[40]	92	6 cycles	Ovulation, pregnancy, abortion, and live-birth rates	MET<br>CC	1700mg/d, continuously<br>150mg/d	26.4<br>25.9	27<br>26.7	1.6<br>1.7
Rouzi AA 2006[41]	25	3 cycles	Ovulation rate, number of follicles, pregnancy rate, and changes in metabolic and endocrine factors	CC+ROS<br><br>CC+MET	CC,150mg/d<br>ROS, 8mg/d, continuously<br>CC,150mg/d<br>MET, 1500mg/d, continuously	28.6<br><br>27.4	30.9<br><br>32	6.47<br><br>5.21
Sheikh-El-Arab 2011[42]	116	1 cycle	Ovulation rate, number of follicles, final endometrial thickness, mid-luteal progesterone, and pregnancy	CC<br>LZ	100mg/d<br>5mg/d	25<br>24.95	29.18<br>27.7	NA<br>NA
Syed 2018[43]	102	3 cycles	Follicular size, ovulation and pregnancy rates	MET<br>PIO	1700mg/d, continuously<br>30mg/d, continuously	28.16<br>28.18	NA<br>NA	NA<br>NA
Vandermolen DT 2001[44]	25	6 cycles	Ovulation and pregnancy rate	CC<br>CC+MET	50-150 mg/d<br>50-150 mg/d<br>MET, 1500mg/d, continuously	30<br>29	38.4<br>37.6	NA<br>NA
Wang 2017[45]	78	9 cycles	Ovulation rate and pregnancy rate	CC+EXE<br><br>CC+MET	CC, 50-150 mg/d<br>EXE, 10-20ug/d, continuously<br>CC, 50-150 mg/d<br>MET, 250-2000mg/d, continuously	25.92<br><br>25.67	26.26<br><br>25.74	NA<br><br>NA
Wang 2021[46]	180	1 cycle	Endometrial receptivity, ovulation rate, pregnancy rates	LZ<br>CC	2.5mg/d<br>50mg/d	28.5<br>28.3	24.9<br>25.3	2.4<br>2.3
			Rates of ovulation, pregnancy and	MET	1500mg/d, continuously	27.8	32.9	NA

Zain 2009[47]	115	6 cycles	live birth	CC	50-200 mg/d	29.6	33.9	NA
				CC+MET	CC,50-200 mg/d<br>MET, 1500mg/d, continuously	29.3	33	NA

Supplementary Table 3. League table of outcomes

Supplementary Table 3A. League table- Clinical pregnancy

CC											
-1.3 (-2.74, 0.1)	EXE										
**-1.76 (-3.3, -0.27)**	-0.46 (-2.49, 1.57)	CC+EXE									
**-0.62 (-1.08, -0.18)**	0.68 (-0.78, 2.18)	1.14 (-0.41, 2.74)	LZ								
-0.07 (-0.65, 0.46)	1.23 (-0.08, 2.53)	**1.69 (0.14, 3.26)**	0.55 (-0.17, 1.23)	MET							
**-0.61 (-1.06, -0.2)**	0.69 (-0.73, 2.12)	1.15 (-0.3, 2.62)	0.01 (-0.6, 0.59)	-0.54 (-1.11, 0.03)	CC+MET						
**-1.62 (-3.06, -0.22)**	-0.31 (-2.29, 1.65)	0.14 (-1.85, 2.14)	-0.99 (-2.49, 0.47)	**-1.54 (-3.02, -0.08)**	-1.01 (-2.37, 0.35)	CC+MET+PIO					
**-1.02 (-1.87, -0.18)**	0.27 (-1.34, 1.94)	0.73 (-0.96, 2.47)	-0.4 (-1.22, 0.42)	-0.95 (-1.93, 0.06)	-0.42 (-1.3, 0.51)	0.59 (-1.01, 2.24)	LZ+MET				
**-1.94 (-3.24, -0.68)**	-0.65 (-2.39, 1.1)	-0.19 (-2.12, 1.77)	-1.33 (-2.69, 0.01)	**-1.87 (-3.04, -0.71)**	**-1.33 (-2.63, -0.05)**	-0.33 (-2.19, 1.55)	-0.92 (-2.45, 0.6)	PIO			
1.2 (-0.02, 2.35)	**2.5 (0.82, 4.15)**	**2.96 (1.07, 4.82)**	**1.82 (0.53, 3.05)**	**1.27 (0.21, 2.3)**	**1.81 (0.6, 2.98)**	**2.82 (0.99, 4.6)**	**2.22 (0.75, 3.64)**	**3.14 (1.56, 4.7)**	Placebo		
-0.43 (-1.6, 0.71)	0.88 (-0.93, 2.69)	1.34 (-0.52, 3.21)	0.2 (-1.04, 1.42)	-0.35 (-1.6, 0.9)	0.19 (-0.99, 1.36)	1.19 (-0.6, 2.99)	0.6 (-0.83, 2)	1.52 (-0.19, 3.23)	-1.62 (-3.24, 0.02)	CC+ROS	
-0.45	0.85	1.31	0.17	-0.38	0.16	1.17	0.57	1.5	-1.65	-0.03	TAM

(-1.72, 0.83)	(-1.03, 2.77)	(-0.64, 3.3)	(-1.08, 1.44)	(-1.74, 1.03)	(-1.15, 1.51)	(-0.72, 3.09)	(-0.9, 2.06)	(-0.3, 3.3)	(-3.34, 0.12)	(-1.73, 1.71)	

Supplementary Table 3B. League table- Live birth

CC								
-0.54 (-1.49, 0.39)	LZ							
0.15 (-0.67, 0.93)	0.7 (-0.52, 1.88)	MET						
-0.43 (-1.15, 0.22)	0.11 (-0.99, 1.16)	-0.59 (-1.44, 0.25)	CC+MET					
-1.46 (-3.4, 0.4)	-0.92 (-3.02, 1.13)	-1.61 (-3.6, 0.34)	-1.02 (-2.81, 0.75)	CC+MET+PIO				
-0.59 (-2.08, 0.89)	-0.04 (-1.6, 1.51)	-0.74 (-2.36, 0.91)	-0.15 (-1.65, 1.39)	0.87 (-1.44, 3.23)	LZ+MET			
0.16 (-2.77, 3.03)	0.7 (-2.35, 3.73)	0.01 (-2.79, 2.79)	0.6 (-2.32, 3.5)	1.62 (-1.78, 5.04)	0.75 (-2.49, 3.95)	PIO		
1.33 (-1.15, 4.04)	1.88 (-0.77, 4.73)	1.18 (-1.16, 3.78)	1.76 (-0.71, 4.5)	2.8 (-0.25, 6.06)	1.92 (-0.95, 4.96)	1.18 (-2.45, 4.98)	Placebo	
-0.96 (-3.42, 1.44)	-0.42 (-3.01, 2.11)	-1.11 (-3.61, 1.35)	-0.52 (-2.87, 1.8)	0.5 (-2.44, 3.42)	-0.37 (-3.17, 2.36)	-1.12 (-4.89, 2.6)	-2.3 (-5.88, 1.09)	CC+ROS

Supplementary Table 3C. League table -Miscarriage

CC								
-0.24<br>(-0.92, 0.57)	LZ							
-0.5<br>(-1.37, 0.1)	-0.27<br>(-1.45, 0.54)	CC+MET						
-0.52<br>(-3.25, 2.07)	-0.29<br>(-3.12, 2.36)	0.01<br>(-2.57, 2.55)	CC+MET+PIO					
-0.18<br>(-1.3, 0.6)	0.06<br>(-1.38, 1.03)	0.33<br>(-0.64, 1.16)	0.32<br>(-2.42, 3.02)	MET				
-0.22<br>(-2.24, 1.5)	0.01<br>(-2.21, 1.83)	0.29<br>(-1.61, 2.05)	0.28<br>(-2.9, 3.37)	-0.03<br>(-1.66, 1.54)	Placebo			
-0.84<br>(-2.16, 0.45)	-0.6<br>(-2.03, 0.69)	-0.33<br>(-1.56, 1.13)	-0.31<br>(-3.13, 2.63)	-0.66<br>(-2.02, 1.06)	-0.62<br>(-2.68, 1.77)	LZ+MET		
-1.66<br>(-5.44, 1.06)	-1.44<br>(-5.31, 1.36)	-1.14<br>(-4.84, 1.6)	-1.18<br>(-5.63, 2.61)	-1.45<br>(-5.02, 1.19)	-1.44<br>(-5.28, 1.69)	-0.84<br>(-4.77, 2.16)	PIO	
-0.42<br>(-4.36, 3.46)	-0.2<br>(-4.2, 3.74)	0.11<br>(-3.75, 3.96)	0.13<br>(-4.54, 4.7)	-0.22<br>(-4.19, 3.76)	-0.18<br>(-4.41, 4.11)	0.42<br>(-3.68, 4.49)	1.33<br>(-3.56, 6.61)	CC+ROS

Supplementary Table 3D. League table -Ectopic pregnancy

CC					
-0.26<br>(-1.71, 1.18)	LZ				
**19.69**<br>**(1.83, 61.71)**	**19.95**<br>**(2, 62.05)**	MET			
-0.3<br>(-2.12, 1.48)	-0.05<br>(-2.25, 2.14)	**-20.05**<br>**(-62.09, -2.12)**	CC+MET		
7.45<br>(-21.62, 53)	7.74<br>(-21.32, 53.2)	-11.25<br>(-33.7, 0.57)	7.8<br>(-21.37, 53.27)	Placebo	
17.26<br>(-0.03, 59.64)	**17.46**<br>**(0.16, 59.8)**	-2.79<br>(-52.12, 44.39)	**17.56**<br>**(0.18, 60)**	10.44<br>(-42.11, 59.56)	LZ+MET

Supplementary Table 3E. League table -Multiple pregnancy

CC						
-0.68<br>(-3.31, 1.24)	LZ					
0.43	1.12	CC+MET				

(-1.25, 2.27)	(-1.4, 4.42)					
1.28<br>(-1.93, 4.69)	1.98<br>(-1.64, 6.43)	0.84<br>(-1.93, 3.7)	CC+MET+PIO			
**20.56**<br>**(3.14, 65.43)**	**21.34**<br>**(3.64, 66.11)**	**20.16**<br>**(2.64, 65.06)**	**19.32**<br>**(1.47, 63.85)**	MET		
**20.76**<br>**(2.43, 65.01)**	**21.57**<br>**(2.99, 65.86)**	**20.34**<br>**(1.93, 64.62)**	**19.47**<br>**(0.74, 63.64)**	0.07<br>(-4.26, 4.34)	Placebo	
-13.35<br>(-46.11, 0.85)	-12.63<br>(-45.41, 1.99)	-13.73<br>(-46.56, 0.2)	**-14.65**<br>**(-47.48, -0.22)**	**-37.65**<br>**(-85.18, -8.35)**	**-37.76**<br>**(-85.14, -8.11)**	CC+ROS

Comparisons between treatments read from left to right with Log odds ratio (OR, 95% confidence interval). Significant estimates are presented in bold.

Supplementary Figure 1. The consistency test of different interventions

A. Clinical pregnancy

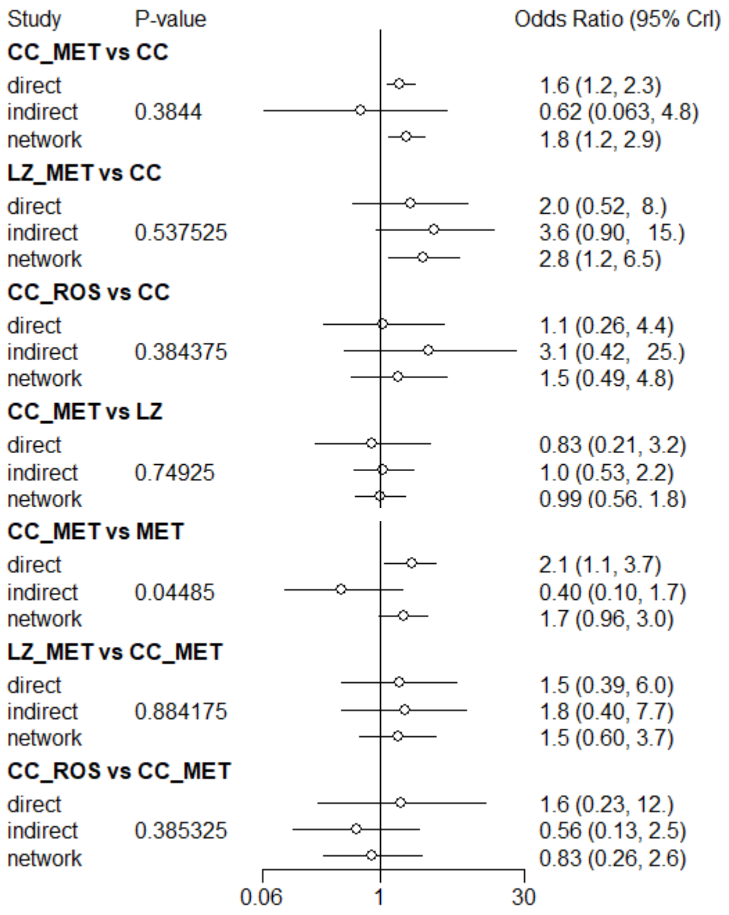

B. Live birth

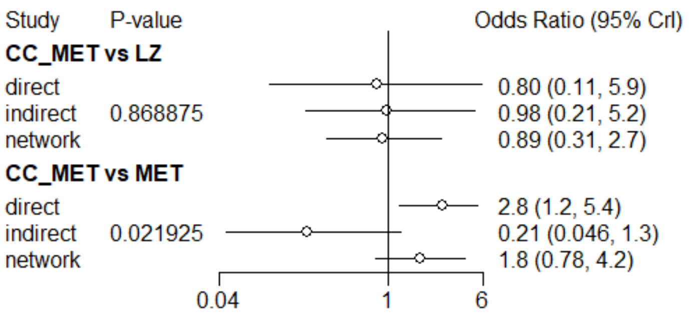

C. Miscarriage

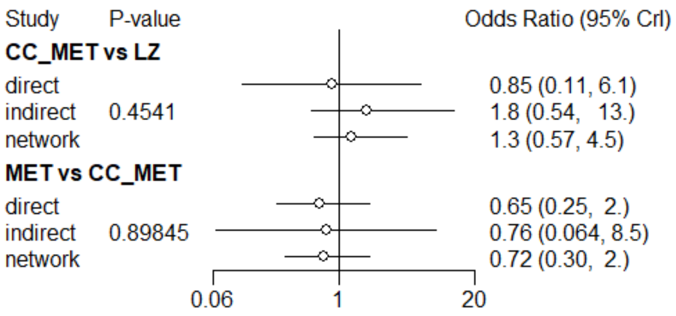

Supplementary Figure 2. The forest of pregnancy rate using frequentist method

A. Clinical pregnancy

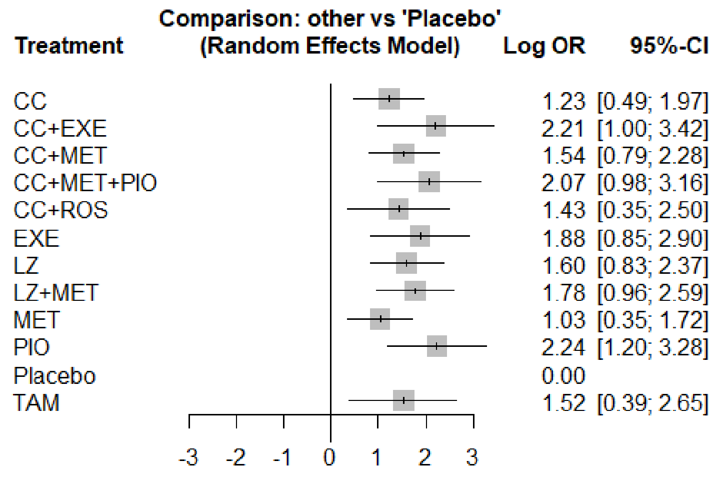

B. Live birth

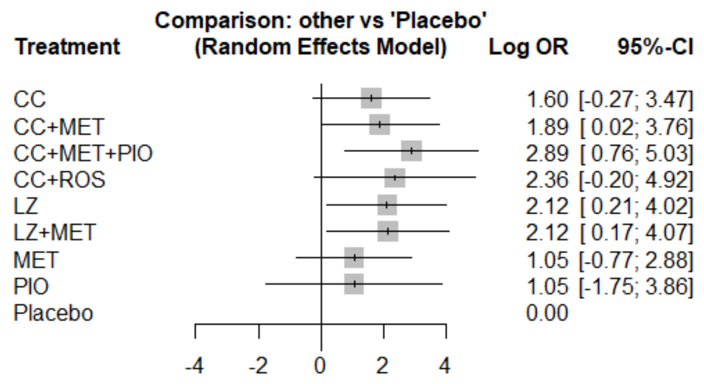

Supplementary Figure 3. The heterogeneity test of different interventions

A. Clinical pregnancy

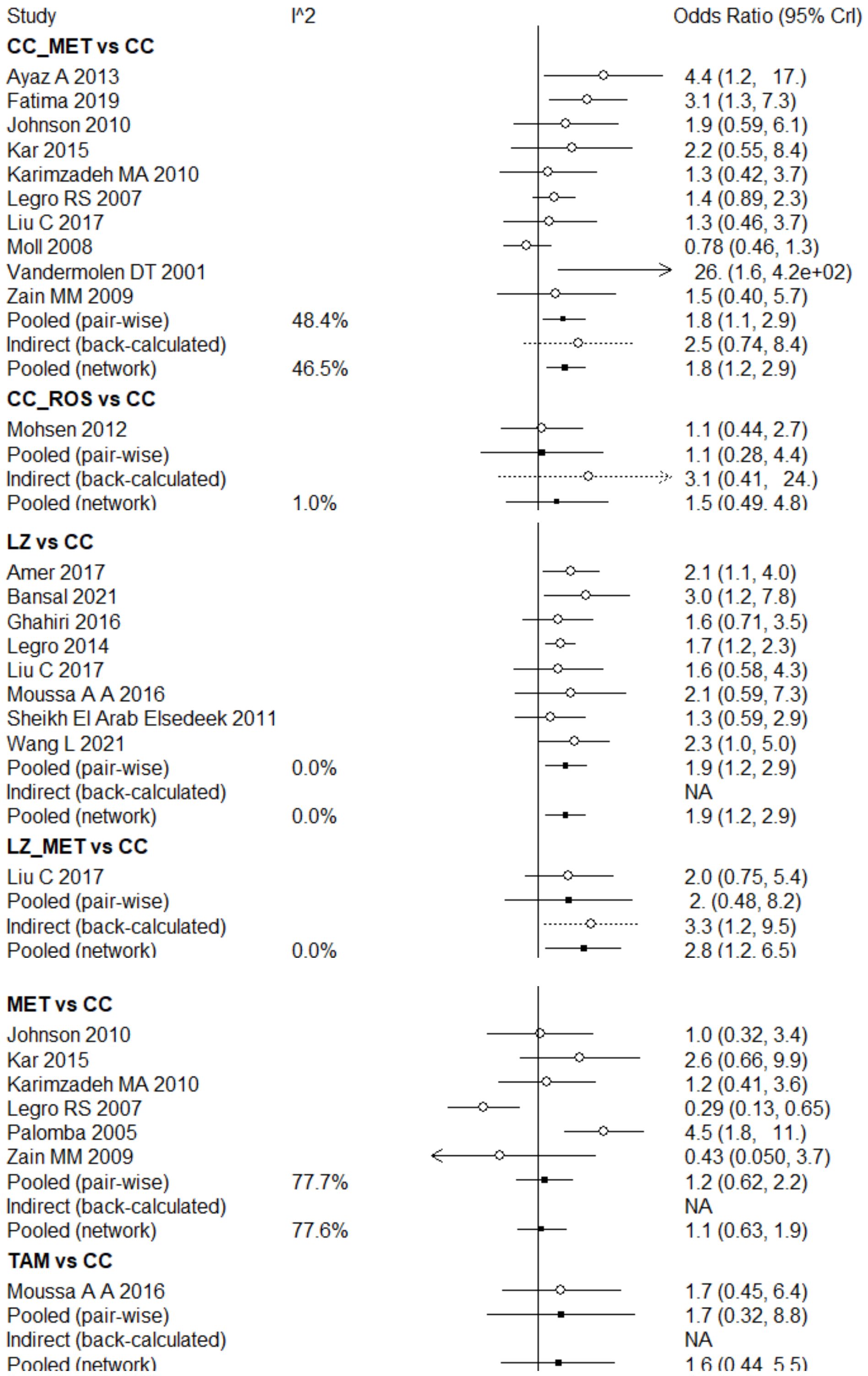

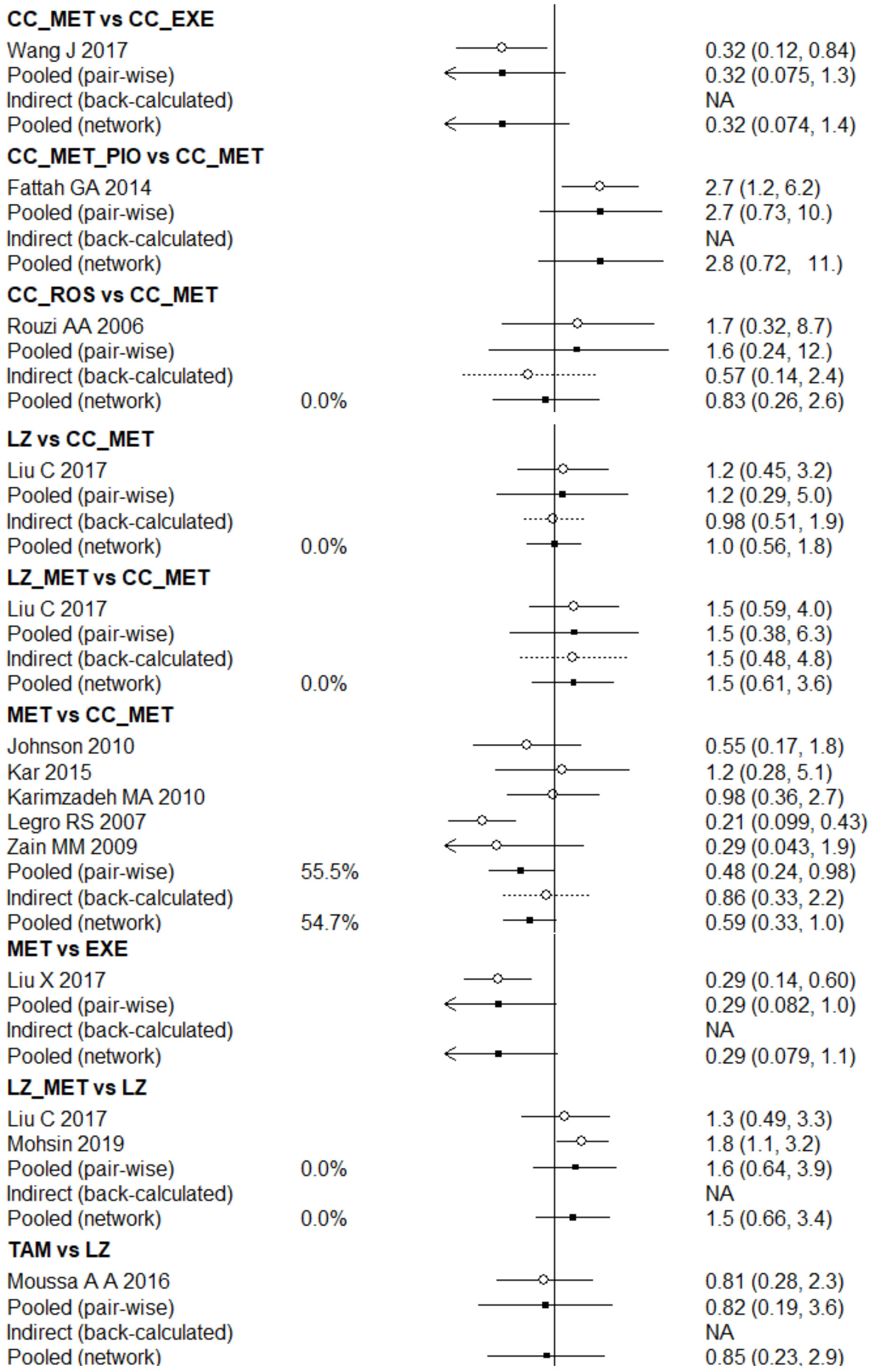
CC_MET vs CC_EXE
Wang J 2017 0.32 (0.12, 0.84)
Pooled (pair-wise) 0.32 (0.075, 1.3)
Indirect (back-calculated) NA
Pooled (network) 0.32 (0.074, 1.4)
CC_MET_PIO vs CC_MET
Fattah GA 2014 2.7 (1.2, 6.2)
Pooled (pair-wise) 2.7 (0.73, 10.)
Indirect (back-calculated) NA
Pooled (network) 2.8 (0.72, 11.)
CC_ROS vs CC_MET
Rouzi AA 2006 1.7 (0.32, 8.7)
Pooled (pair-wise) 1.6 (0.24, 12.)
Indirect (back-calculated) 0.57 (0.14, 2.4)
Pooled (network) 0.0% 0.83 (0.26, 2.6)
LZ vs CC_MET
Liu C 2017 1.2 (0.45, 3.2)
Pooled (pair-wise) 1.2 (0.29, 5.0)
Indirect (back-calculated) 0.98 (0.51, 1.9)
Pooled (network) 0.0% 1.0 (0.56, 1.8)
LZ_MET vs CC_MET
Liu C 2017 1.5 (0.59, 4.0)
Pooled (pair-wise) 1.5 (0.38, 6.3)
Indirect (back-calculated) 1.5 (0.48, 4.8)
Pooled (network) 0.0% 1.5 (0.61, 3.6)
MET vs CC_MET
Johnson 2010 0.55 (0.17, 1.8)
Kar 2015 1.2 (0.28, 5.1)
Karimzadeh MA 2010 0.98 (0.36, 2.7)
Legro RS 2007 0.21 (0.099, 0.43)
Zain MM 2009 0.29 (0.043, 1.9)
Pooled (pair-wise) 55.5% 0.48 (0.24, 0.98)
Indirect (back-calculated) 0.86 (0.33, 2.2)
Pooled (network) 54.7% 0.59 (0.33, 1.0)
MET vs EXE
Liu X 2017 0.29 (0.14, 0.60)
Pooled (pair-wise) 0.29 (0.082, 1.0)
Indirect (back-calculated) NA
Pooled (network) 0.29 (0.079, 1.1)
LZ_MET vs LZ
Liu C 2017 1.3 (0.49, 3.3)
Mohsin 2019 1.8 (1.1, 3.2)
Pooled (pair-wise) 0.0% 1.6 (0.64, 3.9)
Indirect (back-calculated) NA
Pooled (network) 0.0% 1.5 (0.66, 3.4)
TAM vs LZ
Moussa A A 2016 0.81 (0.28, 2.3)
Pooled (pair-wise) 0.82 (0.19, 3.6)
Indirect (back-calculated) NA
Pooled (network) 0.85 (0.23, 2.9)

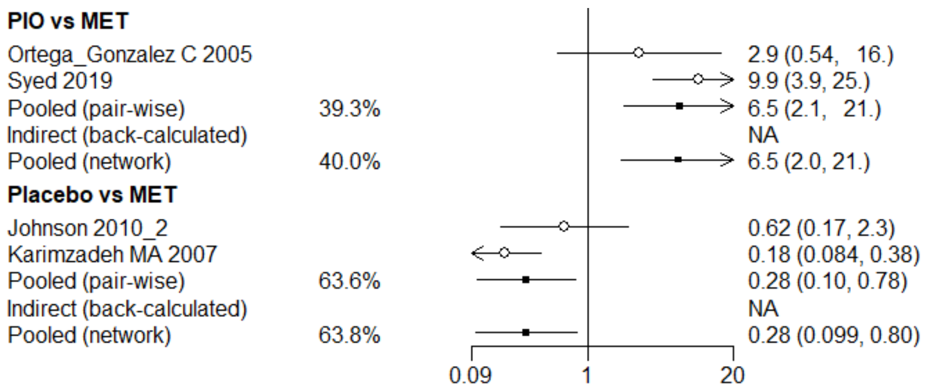
PIO vs MET
Ortega_Gonzalez C 2005 2.9 (0.54, 16.)
Syed 2019 9.9 (3.9, 25.)
Pooled (pair-wise) 39.3% 6.5 (2.1, 21.)
Indirect (back-calculated) NA
Pooled (network) 40.0% 6.5 (2.0, 21.)
Placebo vs MET
Johnson 2010_2 0.62 (0.17, 2.3)
Karimzadeh MA 2007 0.18 (0.084, 0.38)
Pooled (pair-wise) 63.6% 0.28 (0.10, 0.78)
Indirect (back-calculated) NA
Pooled (network) 63.8% 0.28 (0.099, 0.80)
0.09
1
20

B. Live birth

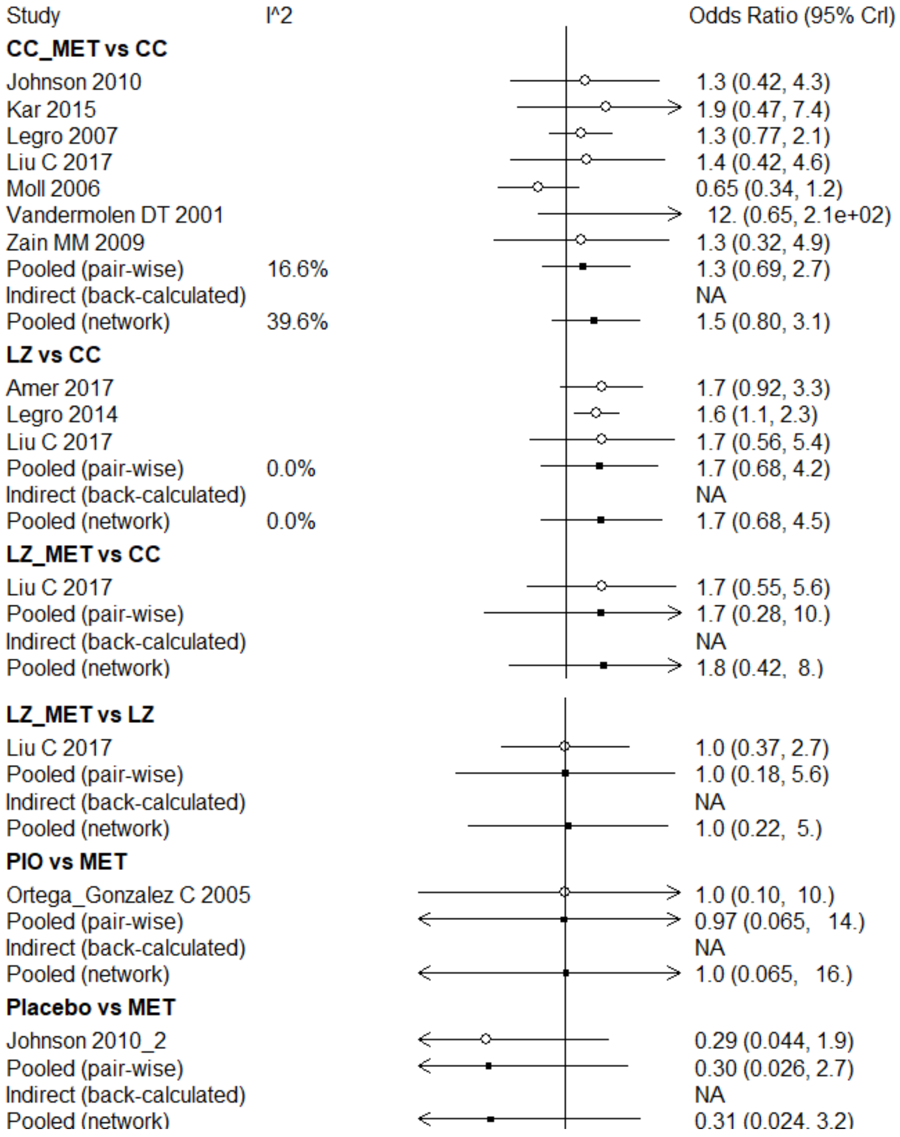
Study
I^2
Odds Ratio (95% CrI)
CC_MET vs CC
Johnson 2010 1.3 (0.42, 4.3)
Kar 2015 1.9 (0.47, 7.4)
Legro 2007 1.3 (0.77, 2.1)
Liu C 2017 1.4 (0.42, 4.6)
Moll 2006 0.65 (0.34, 1.2)
Vandermolen DT 2001 12. (0.65, 2.1e+02)
Zain MM 2009 1.3 (0.32, 4.9)
Pooled (pair-wise) 16.6% 1.3 (0.69, 2.7)
Indirect (back-calculated) NA
Pooled (network) 39.6% 1.5 (0.80, 3.1)
LZ vs CC
Amer 2017 1.7 (0.92, 3.3)
Legro 2014 1.6 (1.1, 2.3)
Liu C 2017 1.7 (0.56, 5.4)
Pooled (pair-wise) 0.0% 1.7 (0.68, 4.2)
Indirect (back-calculated) NA
Pooled (network) 0.0% 1.7 (0.68, 4.5)
LZ_MET vs CC
Liu C 2017 1.7 (0.55, 5.6)
Pooled (pair-wise) 1.7 (0.28, 10.)
Indirect (back-calculated) NA
Pooled (network) 1.8 (0.42, 8.)
LZ_MET vs LZ
Liu C 2017 1.0 (0.37, 2.7)
Pooled (pair-wise) 1.0 (0.18, 5.6)
Indirect (back-calculated) NA
Pooled (network) 1.0 (0.22, 5.)
PIO vs MET
Ortega_Gonzalez C 2005 1.0 (0.10, 10.)
Pooled (pair-wise) 0.97 (0.065, 14.)
Indirect (back-calculated) NA
Pooled (network) 1.0 (0.065, 16.)
Placebo vs MET
Johnson 2010_2 0.29 (0.044, 1.9)
Pooled (pair-wise) 0.30 (0.026, 2.7)
Indirect (back-calculated) NA
Pooled (network) 0.31 (0.024, 3.2)

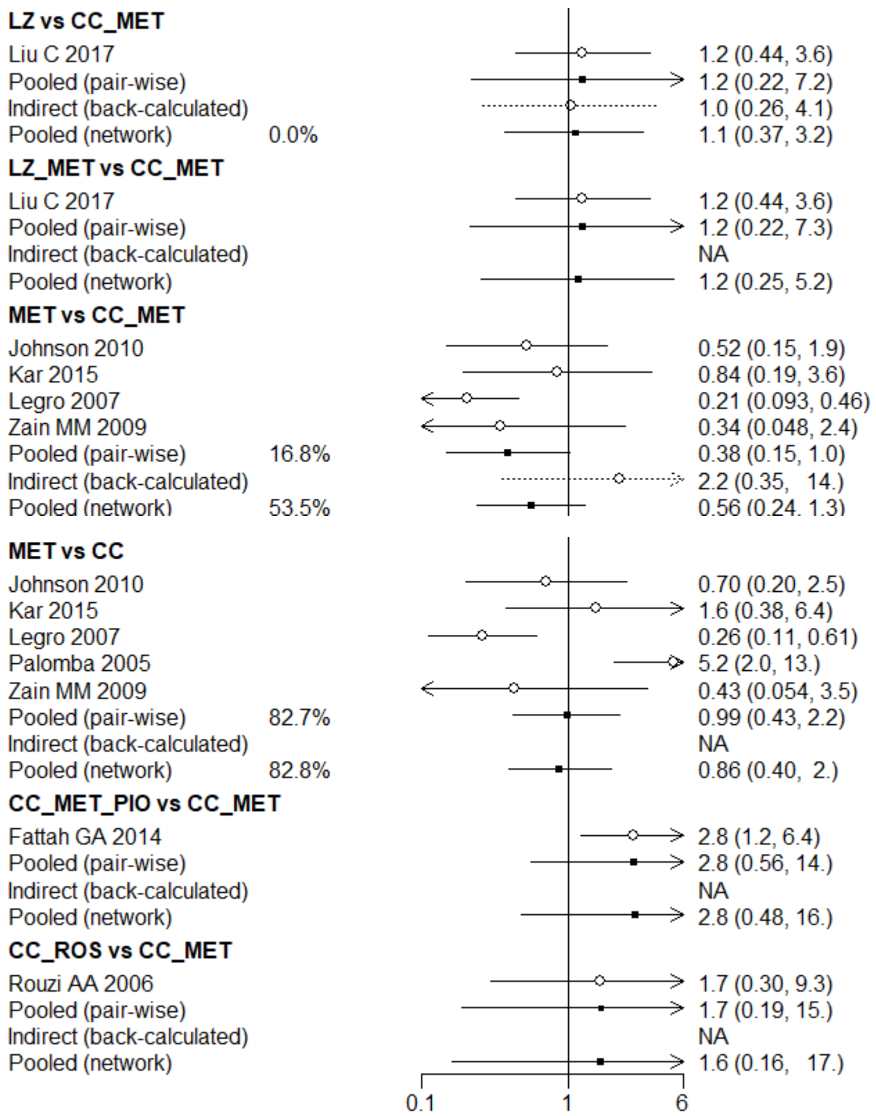

C. Miscarriage

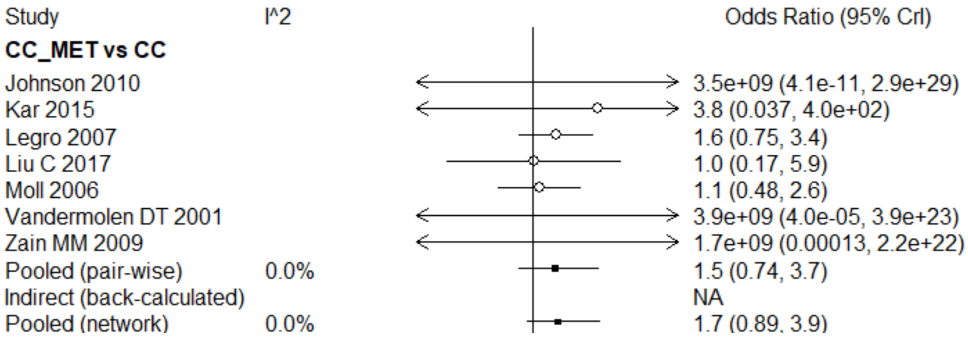

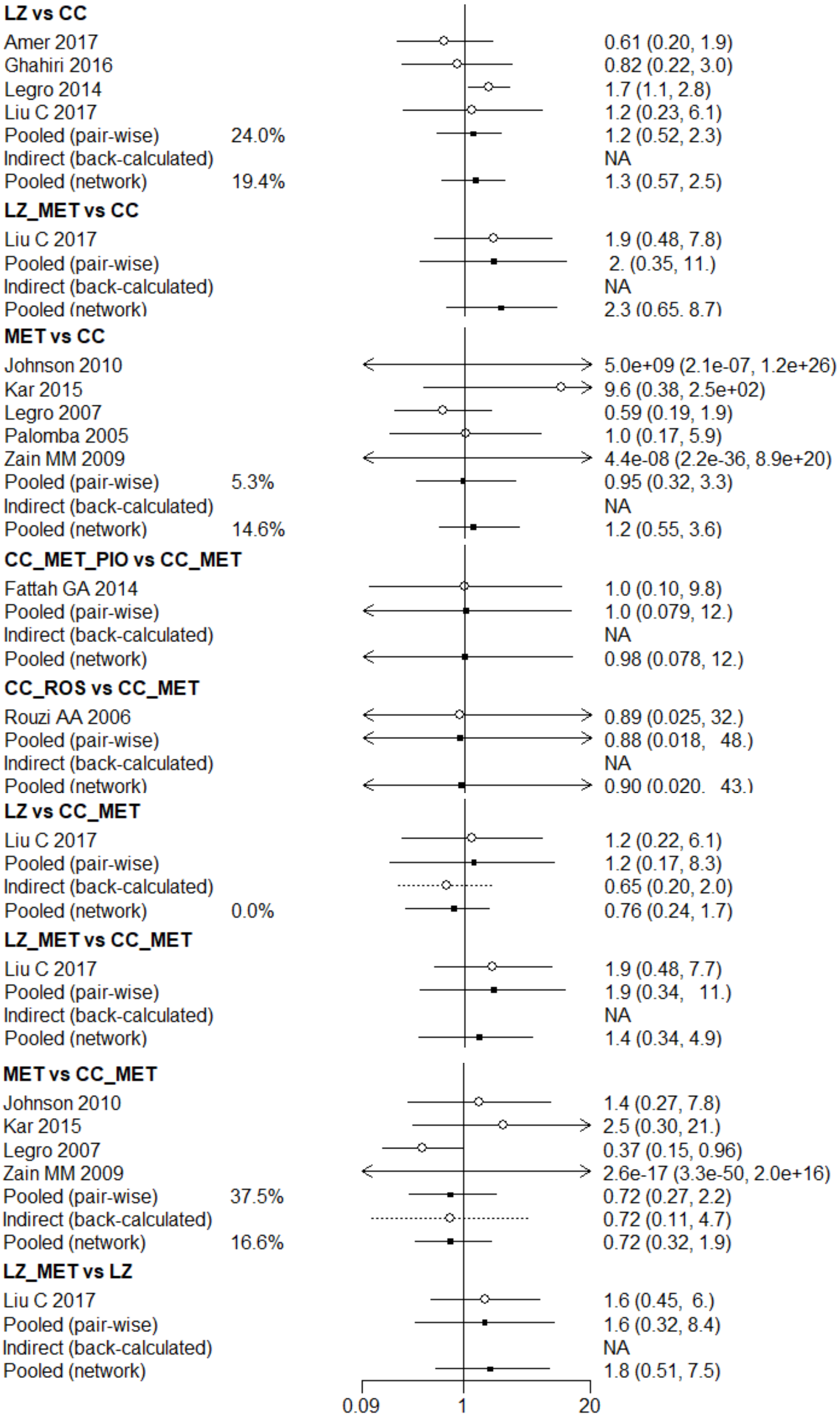

D. Ectopic pregnancy

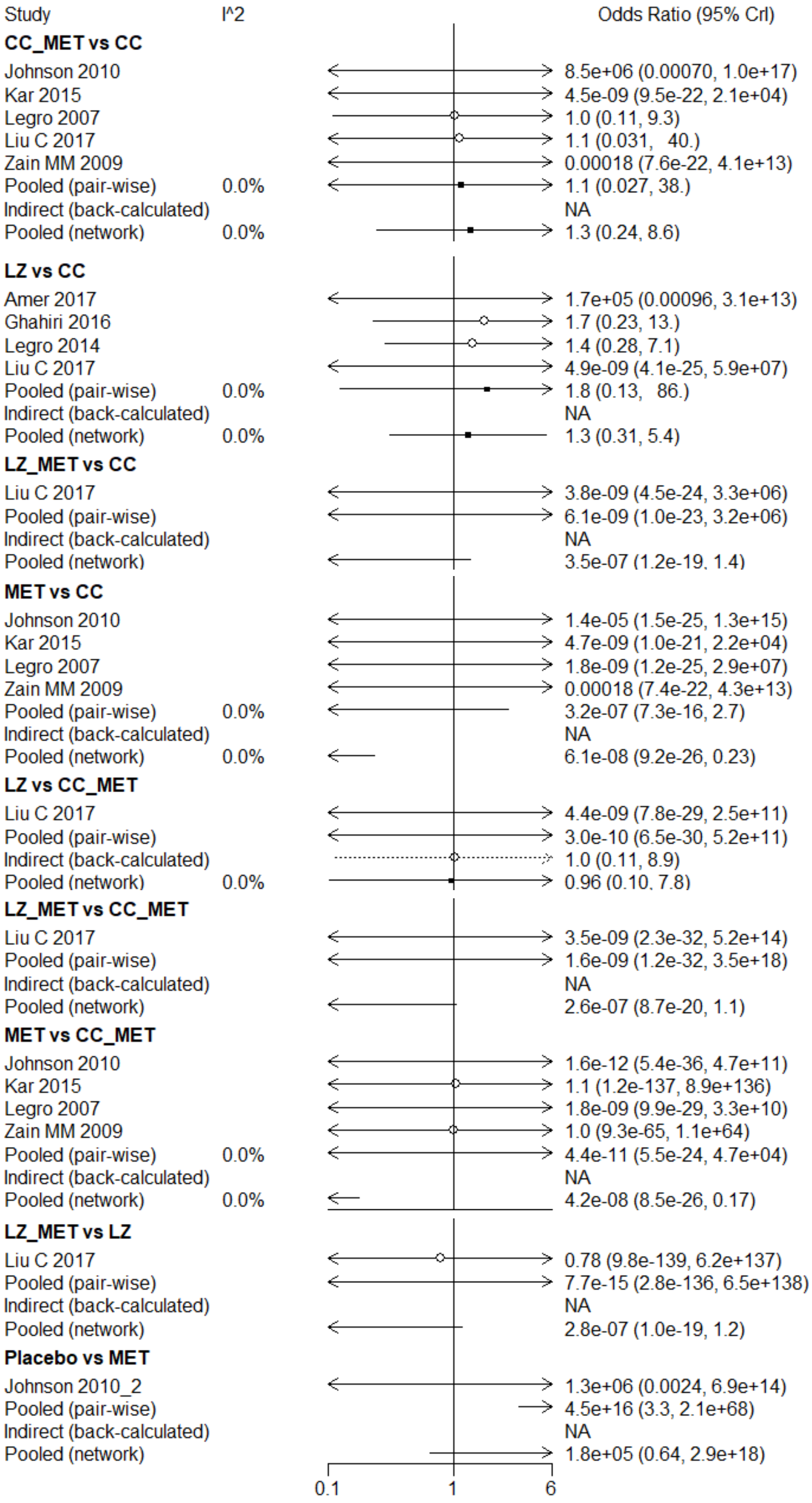
Study
I^2
Odds Ratio (95% CrI)
CC_MET vs CC
Johnson 2010 8.5e+06 (0.00070, 1.0e+17)
Kar 2015 4.5e-09 (9.5e-22, 2.1e+04)
Legro 2007 1.0 (0.11, 9.3)
Liu C 2017 1.1 (0.031, 40.)
Zain MM 2009 0.00018 (7.6e-22, 4.1e+13)
Pooled (pair-wise) 0.0% 1.1 (0.027, 38.)
Indirect (back-calculated) NA
Pooled (network) 0.0% 1.3 (0.24, 8.6)
LZ vs CC
Amer 2017 1.7e+05 (0.00096, 3.1e+13)
Ghahiri 2016 1.7 (0.23, 13.)
Legro 2014 1.4 (0.28, 7.1)
Liu C 2017 4.9e-09 (4.1e-25, 5.9e+07)
Pooled (pair-wise) 0.0% 1.8 (0.13, 86.)
Indirect (back-calculated) NA
Pooled (network) 0.0% 1.3 (0.31, 5.4)
LZ_MET vs CC
Liu C 2017 3.8e-09 (4.5e-24, 3.3e+06)
Pooled (pair-wise) 6.1e-09 (1.0e-23, 3.2e+06)
Indirect (back-calculated) NA
Pooled (network) 3.5e-07 (1.2e-19, 1.4)
MET vs CC
Johnson 2010 1.4e-05 (1.5e-25, 1.3e+15)
Kar 2015 4.7e-09 (1.0e-21, 2.2e+04)
Legro 2007 1.8e-09 (1.2e-25, 2.9e+07)
Zain MM 2009 0.00018 (7.4e-22, 4.3e+13)
Pooled (pair-wise) 0.0% 3.2e-07 (7.3e-16, 2.7)
Indirect (back-calculated) NA
Pooled (network) 0.0% 6.1e-08 (9.2e-26, 0.23)
LZ vs CC_MET
Liu C 2017 4.4e-09 (7.8e-29, 2.5e+11)
Pooled (pair-wise) 3.0e-10 (6.5e-30, 5.2e+11)
Indirect (back-calculated) 1.0 (0.11, 8.9)
Pooled (network) 0.0% 0.96 (0.10, 7.8)
LZ_MET vs CC_MET
Liu C 2017 3.5e-09 (2.3e-32, 5.2e+14)
Pooled (pair-wise) 1.6e-09 (1.2e-32, 3.5e+18)
Indirect (back-calculated) NA
Pooled (network) 2.6e-07 (8.7e-20, 1.1)
MET vs CC_MET
Johnson 2010 1.6e-12 (5.4e-36, 4.7e+11)
Kar 2015 1.1 (1.2e-137, 8.9e+136)
Legro 2007 1.8e-09 (9.9e-29, 3.3e+10)
Zain MM 2009 1.0 (9.3e-65, 1.1e+64)
Pooled (pair-wise) 0.0% 4.4e-11 (5.5e-24, 4.7e+04)
Indirect (back-calculated) NA
Pooled (network) 0.0% 4.2e-08 (8.5e-26, 0.17)
LZ_MET vs LZ
Liu C 2017 0.78 (9.8e-139, 6.2e+137)
Pooled (pair-wise) 7.7e-15 (2.8e-136, 6.5e+138)
Indirect (back-calculated) NA
Pooled (network) 2.8e-07 (1.0e-19, 1.2)
Placebo vs MET
Johnson 2010_2 1.3e+06 (0.0024, 6.9e+14)
Pooled (pair-wise) 4.5e+16 (3.3, 2.1e+68)
Indirect (back-calculated) NA
Pooled (network) 1.8e+05 (0.64, 2.9e+18)
0.1
1
6

C.

E. Multiple pregnancy

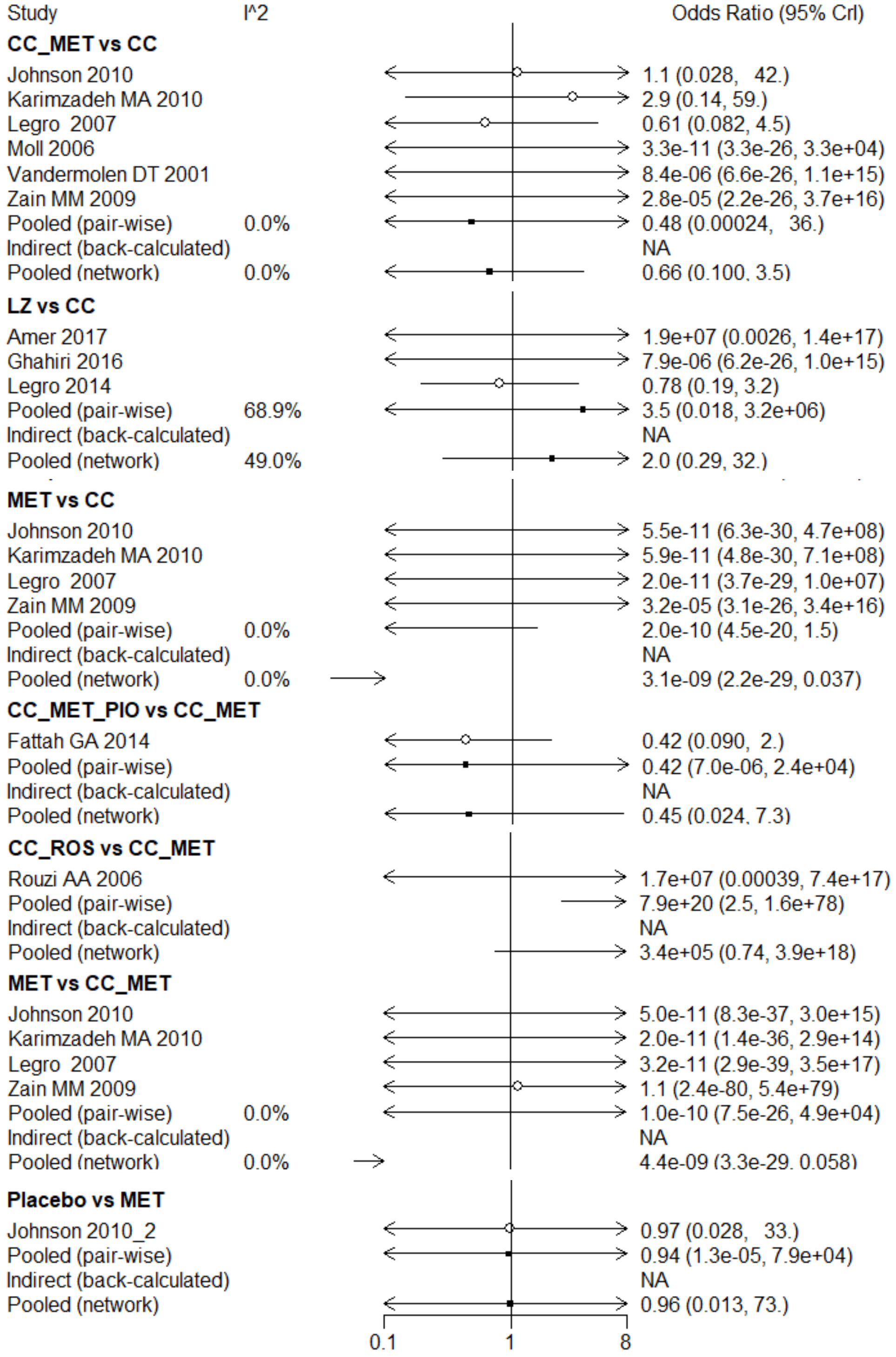

F. Clinical pregnancy in non-obese subgroup

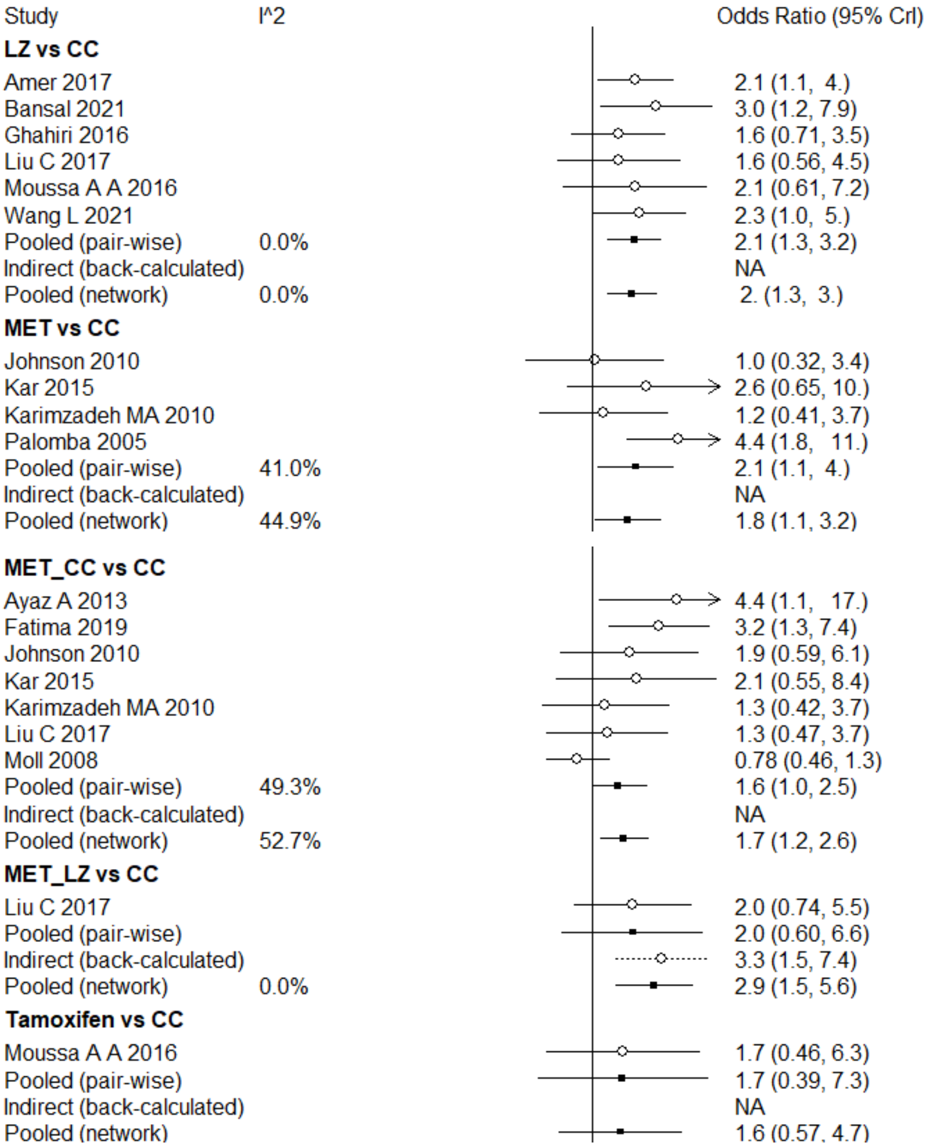

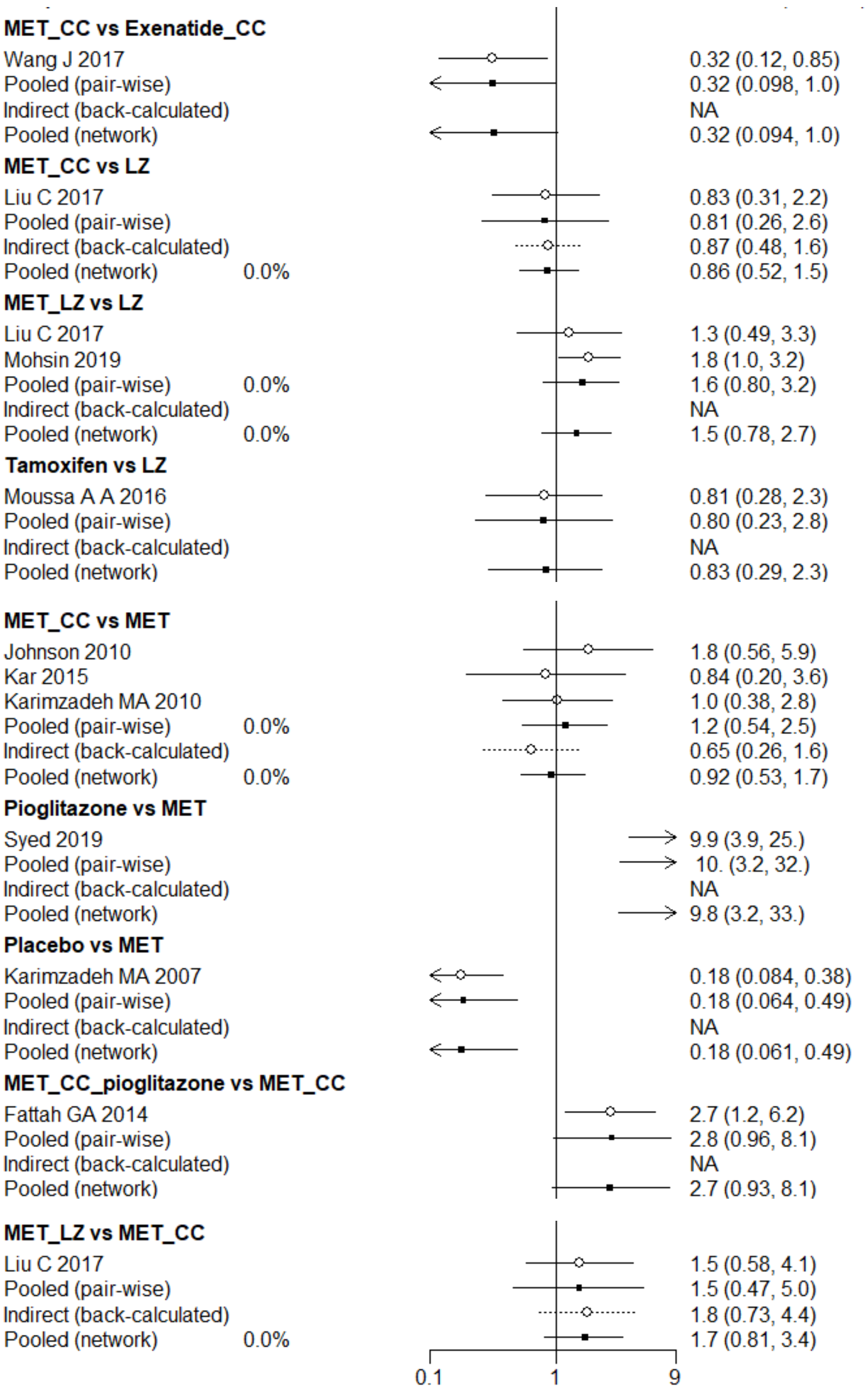

MET_CC vs Exenatide_CC
Wang J 2017 0.32 (0.12, 0.85)
Pooled (pair-wise) 0.32 (0.098, 1.0)
Indirect (back-calculated) NA
Pooled (network) 0.32 (0.094, 1.0)
MET_CC vs LZ
Liu C 2017 0.83 (0.31, 2.2)
Pooled (pair-wise) 0.81 (0.26, 2.6)
Indirect (back-calculated) 0.87 (0.48, 1.6)
Pooled (network) 0.0% 0.86 (0.52, 1.5)
MET_LZ vs LZ
Liu C 2017 1.3 (0.49, 3.3)
Mohsin 2019 1.8 (1.0, 3.2)
Pooled (pair-wise) 0.0% 1.6 (0.80, 3.2)
Indirect (back-calculated) NA
Pooled (network) 0.0% 1.5 (0.78, 2.7)
Tamoxifen vs LZ
Moussa A A 2016 0.81 (0.28, 2.3)
Pooled (pair-wise) 0.80 (0.23, 2.8)
Indirect (back-calculated) NA
Pooled (network) 0.83 (0.29, 2.3)
MET_CC vs MET
Johnson 2010 1.8 (0.56, 5.9)
Kar 2015 0.84 (0.20, 3.6)
Karimzadeh MA 2010 1.0 (0.38, 2.8)
Pooled (pair-wise) 0.0% 1.2 (0.54, 2.5)
Indirect (back-calculated) 0.65 (0.26, 1.6)
Pooled (network) 0.0% 0.92 (0.53, 1.7)
Pioglitazone vs MET
Syed 2019 9.9 (3.9, 25.)
Pooled (pair-wise) 10. (3.2, 32.)
Indirect (back-calculated) NA
Pooled (network) 9.8 (3.2, 33.)
Placebo vs MET
Karimzadeh MA 2007 0.18 (0.084, 0.38)
Pooled (pair-wise) 0.18 (0.064, 0.49)
Indirect (back-calculated) NA
Pooled (network) 0.18 (0.061, 0.49)
MET_CC_pioglitazone vs MET_CC
Fattah GA 2014 2.7 (1.2, 6.2)
Pooled (pair-wise) 2.8 (0.96, 8.1)
Indirect (back-calculated) NA
Pooled (network) 2.7 (0.93, 8.1)
MET_LZ vs MET_CC
Liu C 2017 1.5 (0.58, 4.1)
Pooled (pair-wise) 1.5 (0.47, 5.0)
Indirect (back-calculated) 1.8 (0.73, 4.4)
Pooled (network) 0.0% 1.7 (0.81, 3.4)
0.1
1
9

G. Clinical pregnancy in obese subgroup

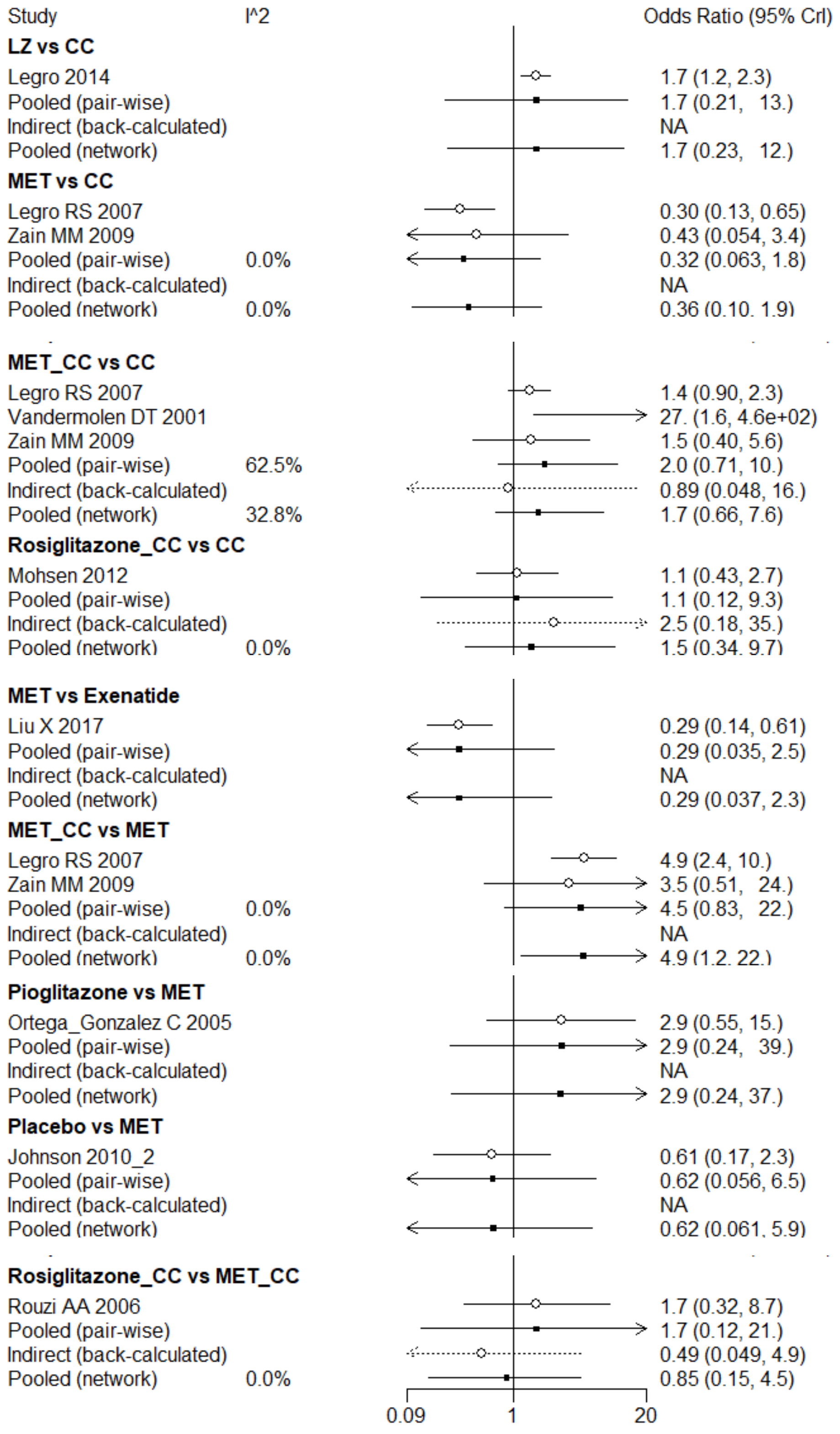

Supplementary Table 4. Network meta-regression analysis of outcomes

Variables	Coef.	Std. Err.	t	P>|t|	95% Conf. Interval	
**Clinical pregnancy**						
BMI	-0.37807	0.252176	-1.5	0.142	-0.88814	0.132003
Region	0.107097	0.23186	0.46	0.647	-0.36188	0.576078
Age	0.2303	0.219201	1.05	0.3	-0.21308	0.673676
Duration of infertility	0.044453	0.224389	0.2	0.844	-0.40942	0.498321
Longest treatment	0.071821	0.215725	0.33	0.741	-0.36452	0.508166
**Live birth**						
BMI	-0.19053	0.392355	-0.49	0.632	-1.00648	0.625418
Region	0.791921	0.536357	1.48	0.155	-0.32349	1.907336
Age	1.152638	0.605761	1.9	0.071	-0.10711	2.412387
Duration of infertility	0.464411	0.49621	0.94	0.36	-0.56751	1.496335
Longest treatment	-0.07314	0.454702	-0.16	0.874	-1.01875	0.87246

Supplementary Figure 4. Forest plot for Subgroup analysis of pregnancy

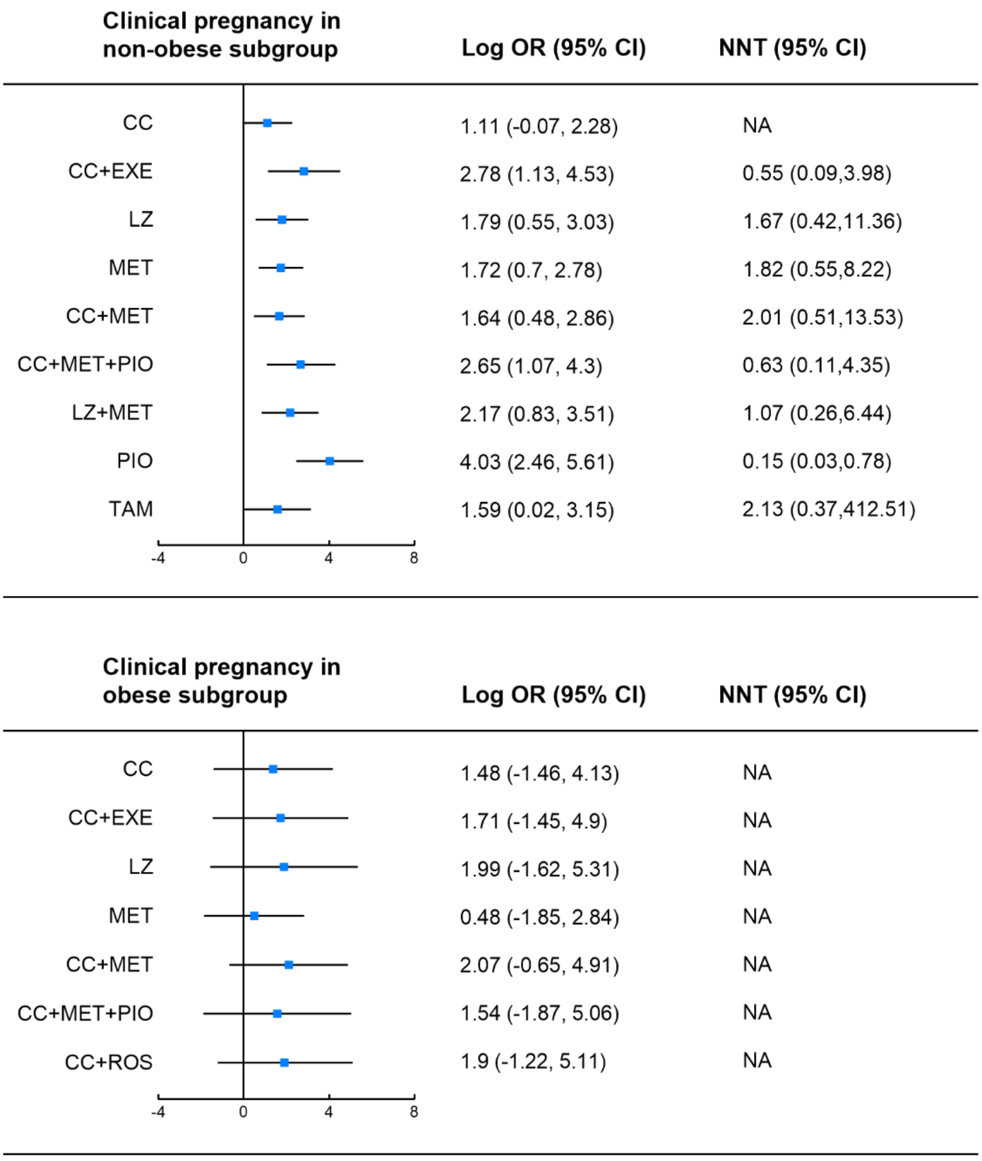

Supplementary Figure 5. Risk of bias assessment in the RCTs

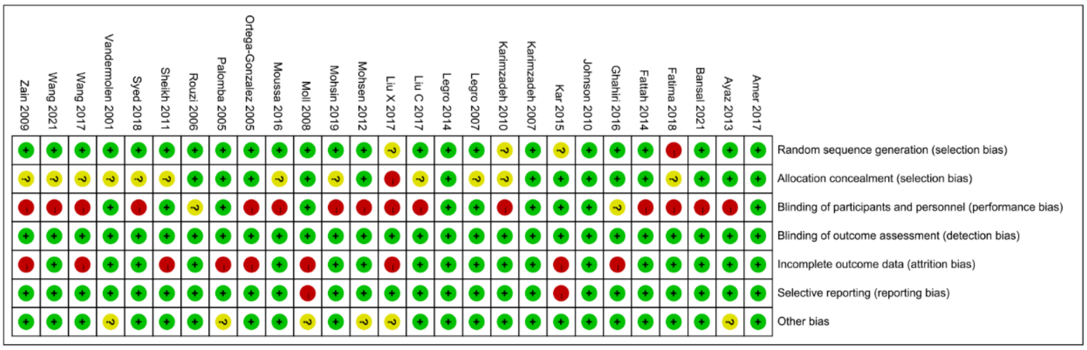

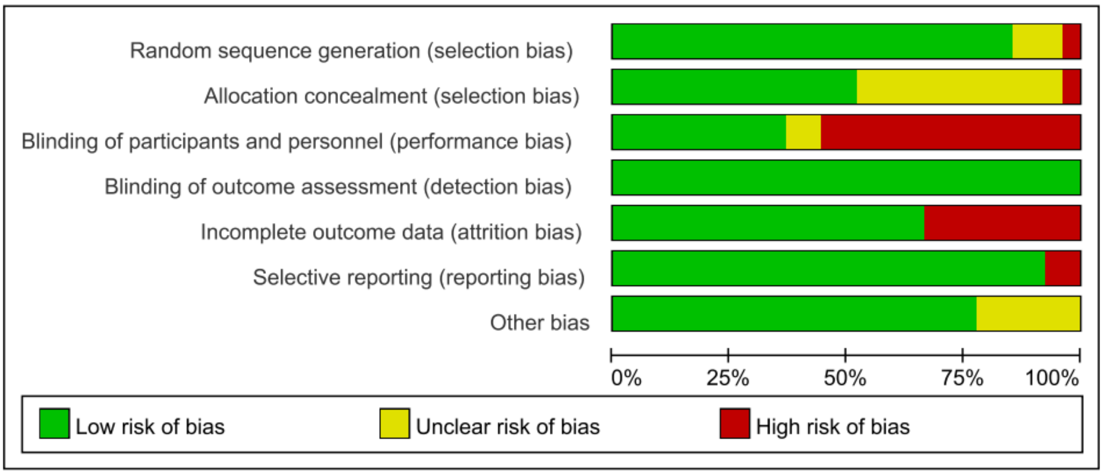

Supplementary Figure 6. The adjusted-funnel plot of primary outcome

A. Clinical pregnancy

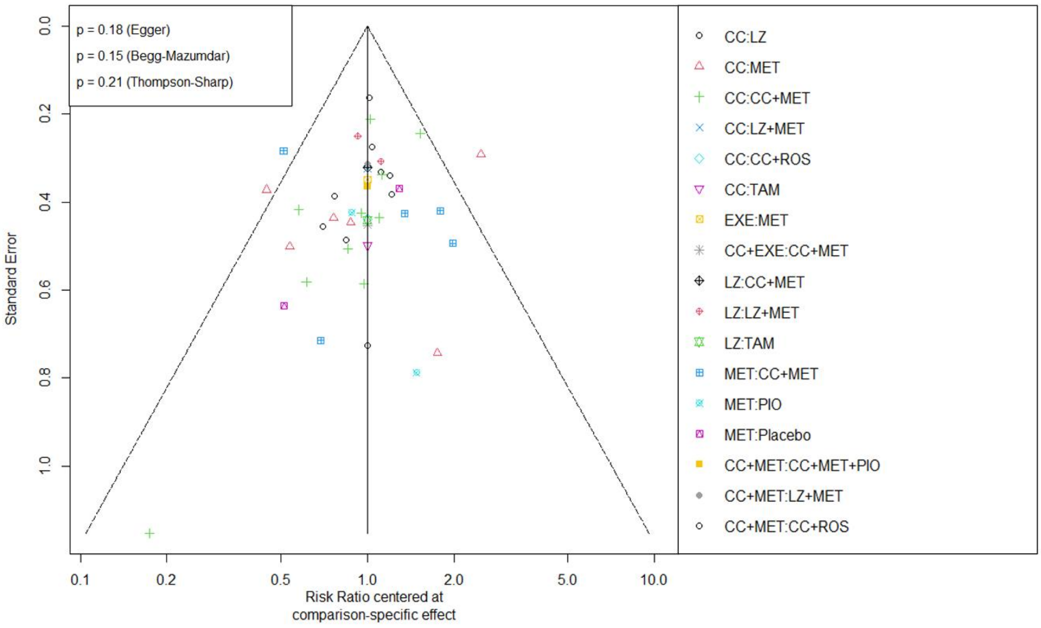

B. Live birth

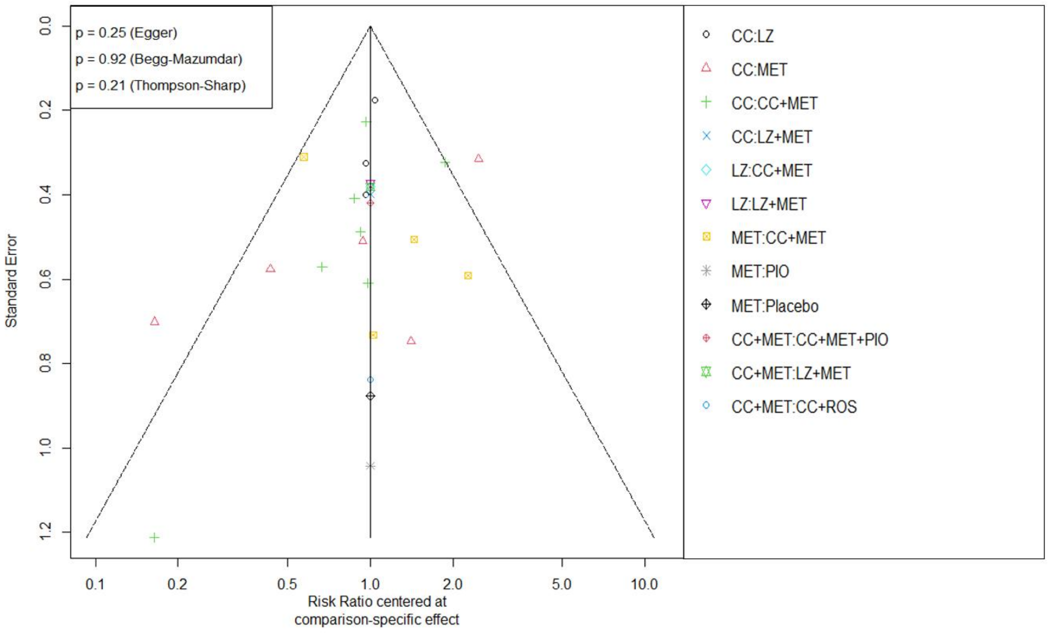

Supplementary Table 5. Confidence in effect estimates

A. Grade of clinical pregnancy

Comparison	Number of studies	Within-study bias	Reporting bias	Indirectness	Imprecision	Heterogeneity	Incoherence	Confidence rating	Reason(s) for downgrading
MET:Placebo	2	Some concerns	Low risk	No concerns	No concerns	Some concerns	No concerns	Low	["Within-study bias","Heterogeneity"]
CC:Placebo	0	Some concerns	Low risk	No concerns	No concerns	Major concerns	No concerns	Very low	["Within-study bias","Heterogeneity"]
CC+EXE:Placebo	0	Some concerns	Low risk	No concerns	No concerns	No concerns	No concerns	Moderate	["Within-study bias"]
CC+MET:Placebo	0	Some concerns	Low risk	No concerns	No concerns	No concerns	No concerns	Moderate	["Within-study bias"]
CC+MET+PIO:Placebo	0	Some concerns	Low risk	No concerns	No concerns	No concerns	No concerns	Moderate	["Within-study bias"]
CC+ROS:Placebo	0	Some concerns	Low risk	No concerns	No concerns	Major concerns	No concerns	Very low	["Within-study bias","Heterogeneity"]
EXE:Placebo	0	Some	Low risk	No concerns	No concerns	No concerns	No concerns	Moderate	["Within-study

		concerns							bias"]
LZ:Placebo	0	Some concerns	Low risk	No concerns	No concerns	No concerns	No concerns	Moderate	["Within-study bias"]
LZ+MET:Placebo	0	Some concerns	Low risk	No concerns	No concerns	No concerns	No concerns	Moderate	["Within-study bias"]
PIO:Placebo	0	Some concerns	Low risk	No concerns	No concerns	No concerns	No concerns	Moderate	["Within-study bias"]
Placebo:TAM	0	Some concerns	Low risk	No concerns	No concerns	Major concerns	No concerns	Very low	["Within-study bias","Heterogeneity"]
CC:CC+MET	10	Some concerns	Low risk	No concerns	No concerns	Major concerns	No concerns	Very low	["Within-study bias","Heterogeneity"]
CC:CC+ROS	1	Some concerns	Low risk	No concerns	Major concerns	No concerns	No concerns	Very low	["Within-study bias","Imprecision"]
CC:LZ	8	Some concerns	Low risk	No concerns	No concerns	Major concerns	No concerns	Very low	["Within-study bias","Heterogeneity"]
CC:LZ+MET	1	Some concerns	Low risk	No concerns	No concerns	Some concerns	No concerns	Low	["Within-study bias","Heterogeneity"]
CC:MET	6	Some concerns	Low risk	No concerns	Major concerns	No concerns	No concerns	Very low	["Within-study bias","Imprecision"]
CC:TAM	1	Some	Low risk	No concerns	Major	No concerns	No concerns	Very low	["Within-study

		concerns			concerns				bias","Imprecision"]
CC+EXE:CC+MET	1	Some concerns	Low risk	No concerns	Some concerns	Some concerns	No concerns	Very low	["Within-study bias","Imprecision","Heterogeneity"]
CC+MET:CC+MET+PIO	1	Some concerns	Low risk	No concerns	Some concerns	Some concerns	No concerns	Very low	["Within-study bias","Imprecision","Heterogeneity"]
CC+MET:CC+ROS	1	Some concerns	Low risk	No concerns	Major concerns	No concerns	No concerns	Very low	["Within-study bias","Imprecision"]
CC+MET:LZ	1	Some concerns	Low risk	No concerns	Major concerns	No concerns	No concerns	Very low	["Within-study bias","Imprecision"]
CC+MET:LZ+MET	1	Some concerns	Low risk	No concerns	Major concerns	No concerns	No concerns	Very low	["Within-study bias","Imprecision"]
CC+MET:MET	5	Some concerns	Low risk	No concerns	Some concerns	Some concerns	No concerns	Very low	["Within-study bias","Imprecision","Heterogeneity"]
EXE:MET	1	Major concerns	Low risk	No concerns	No concerns	Major concerns	No concerns	Very low	["Within-study bias","Heterogeneity"]

LZ:LZ+MET	2	Some concerns	Low risk	No concerns	Major concerns	No concerns	No concerns	Very low	["Within-study bias","Imprecision"]
LZ:TAM	1	Some concerns	Low risk	No concerns	Major concerns	No concerns	No concerns	Very low	["Within-study bias","Imprecision"]
MET:PIO	2	Some concerns	Low risk	No concerns	No concerns	No concerns	No concerns	Moderate	["Within-study bias"]
CC:CC+EXE	0	Some concerns	Low risk	No concerns	No concerns	Some concerns	No concerns	Low	["Within-study bias","Heterogeneity"]
CC:CC+MET+PIO	0	Some concerns	Low risk	No concerns	No concerns	Some concerns	No concerns	Low	["Within-study bias","Heterogeneity"]
CC:EXE	0	Some concerns	Low risk	No concerns	No concerns	Major concerns	No concerns	Very low	["Within-study bias","Heterogeneity"]
CC:PIO	0	Some concerns	Low risk	No concerns	No concerns	No concerns	No concerns	Moderate	["Within-study bias"]
CC+EXE:CC+MET+PIO	0	Some concerns	Low risk	No concerns	Major concerns	No concerns	No concerns	Very low	["Within-study bias","Imprecision"]
CC+EXE:CC+ROS	0	Some concerns	Low risk	No concerns	Major concerns	No concerns	No concerns	Very low	["Within-study bias","Imprecision"]
CC+EXE:EXE	0	Some	Low risk	No concerns	Major	No concerns	No concerns	Very low	["Within-study

		concerns			concerns				bias","Imprecision"]
CC+EXE:LZ	0	Some concerns	Low risk	No concerns	Major concerns	No concerns	No concerns	Very low	["Within-study bias","Imprecision"]
CC+EXE:LZ+MET	0	Some concerns	Low risk	No concerns	Major concerns	No concerns	No concerns	Very low	["Within-study bias","Imprecision"]
CC+EXE:MET	0	Some concerns	Low risk	No concerns	No concerns	Some concerns	No concerns	Low	["Within-study bias","Heterogeneity"]
CC+EXE:PIO	0	Some concerns	Low risk	No concerns	Major concerns	No concerns	No concerns	Very low	["Within-study bias","Imprecision"]
CC+EXE:TAM	0	Some concerns	Low risk	No concerns	Major concerns	No concerns	No concerns	Very low	["Within-study bias","Imprecision"]
CC+MET:EXE	0	Some concerns	Low risk	No concerns	Major concerns	No concerns	No concerns	Very low	["Within-study bias","Imprecision"]
CC+MET:PIO	0	Some concerns	Low risk	No concerns	No concerns	Major concerns	No concerns	Very low	["Within-study bias","Heterogeneity"]
CC+MET:TAM	0	Some concerns	Low risk	No concerns	Major concerns	No concerns	No concerns	Very low	["Within-study bias","Imprecision"]

CC+MET+PIO:CC+ROS	0	Some concerns	Low risk	No concerns	Major concerns	No concerns	No concerns	Very low	["Within-study bias","Imprecision"]
CC+MET+PIO:EXE	0	Some concerns	Low risk	No concerns	Major concerns	No concerns	No concerns	Very low	["Within-study bias","Imprecision"]
CC+MET+PIO:LZ	0	Some concerns	Low risk	No concerns	Major concerns	No concerns	No concerns	Very low	["Within-study bias","Imprecision"]
CC+MET+PIO:LZ+MET	0	Some concerns	Low risk	No concerns	Major concerns	No concerns	No concerns	Very low	["Within-study bias","Imprecision"]
CC+MET+PIO:MET	0	Some concerns	Low risk	No concerns	No concerns	Some concerns	No concerns	Low	["Within-study bias","Heterogeneity"]
CC+MET+PIO:PIO	0	Some concerns	Low risk	No concerns	Major concerns	No concerns	No concerns	Very low	["Within-study bias","Imprecision"]
CC+MET+PIO:TAM	0	Some concerns	Low risk	No concerns	Major concerns	No concerns	No concerns	Very low	["Within-study bias","Imprecision"]
CC+ROS:EXE	0	Some concerns	Low risk	No concerns	Major concerns	No concerns	No concerns	Very low	["Within-study bias","Imprecision"]
CC+ROS:LZ	0	Some concerns	Low risk	No concerns	Major concerns	No concerns	No concerns	Very low	["Within-study bias","Imprecisio

									n"]
CC+ROS:LZ+MET	0	Some concerns	Low risk	No concerns	Major concerns	No concerns	No concerns	Very low	["Within-study bias","Imprecision"]
CC+ROS:MET	0	Some concerns	Low risk	No concerns	Major concerns	No concerns	No concerns	Very low	["Within-study bias","Imprecision"]
CC+ROS:PIO	0	Some concerns	Low risk	No concerns	Some concerns	Some concerns	No concerns	Very low	["Within-study bias","Imprecision","Heterogeneity"]
CC+ROS:TAM	0	Some concerns	Low risk	No concerns	Major concerns	No concerns	No concerns	Very low	["Within-study bias","Imprecision"]
EXE:LZ	0	Some concerns	Low risk	No concerns	Major concerns	No concerns	No concerns	Very low	["Within-study bias","Imprecision"]
EXE:LZ+MET	0	Some concerns	Low risk	No concerns	Major concerns	No concerns	No concerns	Very low	["Within-study bias","Imprecision"]
EXE:PIO	0	Major concerns	Low risk	No concerns	Major concerns	No concerns	No concerns	Very low	["Within-study bias","Imprecision"]
EXE:TAM	0	Some concerns	Low risk	No concerns	Major concerns	No concerns	No concerns	Very low	["Within-study bias","Imprecision"]

LZ:MET	0	Some concerns	Low risk	No concerns	Some concerns	Some concerns	No concerns	Very low	["Within-study bias","Imprecision","Heterogeneity"]
LZ:PIO	0	Some concerns	Low risk	No concerns	No concerns	Major concerns	No concerns	Very low	["Within-study bias","Heterogeneity"]
LZ+MET:MET	0	Some concerns	Low risk	No concerns	No concerns	Major concerns	No concerns	Very low	["Within-study bias","Heterogeneity"]
LZ+MET:PIO	0	Some concerns	Low risk	No concerns	Major concerns	No concerns	No concerns	Very low	["Within-study bias","Imprecision"]
LZ+MET:TAM	0	Some concerns	Low risk	No concerns	Major concerns	No concerns	No concerns	Very low	["Within-study bias","Imprecision"]
MET:TAM	0	Some concerns	Low risk	No concerns	Major concerns	No concerns	No concerns	Very low	["Within-study bias","Imprecision"]
PIO:TAM	0	Some concerns	Low risk	No concerns	Some concerns	Some concerns	No concerns	Very low	["Within-study bias","Imprecision","Heterogeneity"]

B. Grade of clinical pregnancy

Comparison	Number of studies	Within-study bias	Reporting bias	Indirectness	Imprecision	Heterogeneity	Incoherence	Confidence rating	Reason(s) for downgrading
MET:Placebo	1	No concerns	Low risk	No concerns	Major concerns	No concerns	Some concerns	Very low	["Imprecision","Incoherence"]
CC:Placebo	0	No concerns	Low risk	No concerns	Major concerns	No concerns	Some concerns	Very low	["Imprecision","Incoherence"]
CC+MET:Placebo	0	No concerns	Low risk	No concerns	Major concerns	No concerns	Some concerns	Very low	["Imprecision","Incoherence"]
CC+MET+PIO:Placebo	0	Some concerns	Low risk	No concerns	No concerns	Major concerns	Some concerns	Very low	["Within-study bias","Heterogeneity","Incoherence"]
CC+ROS:Placebo	0	No concerns	Low risk	No concerns	Major concerns	No concerns	Some concerns	Very low	["Imprecision","Incoherence"]
LZ:Placebo	0	No concerns	Low risk	No concerns	Major concerns	No concerns	Some concerns	Very low	["Imprecision","Incoherence"]
LZ+MET:Placebo	0	Some concerns	Low risk	No concerns	Major concerns	No concerns	Some concerns	Very low	["Within-study bias","Imprecision","Incoherence"]
PIO:Placebo	0	Some concerns	Low risk	No concerns	Major concerns	No concerns	Some concerns	Very low	["Within-study bias","Imprecision","Incoherence"]

CC:CC+MET	7	Some concerns	Low risk	No concerns	Some concerns	Some concerns	Some concerns	Very low	["Within-study bias","Imprecision","Heterogeneity","Incoherence"]
CC:LZ	3	No concerns	Low risk	No concerns	Some concerns	Some concerns	No concerns	Low	["Imprecision"," Heterogeneity"]
CC:LZ+MET	1	Some concerns	Low risk	No concerns	Major concerns	No concerns	No concerns	Very low	["Within-study bias","Imprecision"]
CC:MET	5	Some concerns	Low risk	No concerns	Major concerns	No concerns	No concerns	Very low	["Within-study bias","Imprecision"]
CC+MET:CC+MET+PIO	1	Some concerns	Low risk	No concerns	Major concerns	No concerns	Some concerns	Very low	["Within-study bias","Imprecision","Incoherence"]
CC+MET:CC+ROS	1	No concerns	Low risk	No concerns	Major concerns	No concerns	Some concerns	Very low	["Imprecision","Incoherence"]
CC+MET:LZ	1	Some concerns	Low risk	No concerns	Major concerns	No concerns	No concerns	Very low	["Within-study bias","Imprecision"]
CC+MET:LZ+MET	1	Some concerns	Low risk	No concerns	Major concerns	No concerns	No concerns	Very low	["Within-study bias","Imprecision"]
CC+MET:MET	4	Some	Low risk	No concerns	Some	Some concerns	Some concerns	Very low	["Within-study

		concerns			concerns				bias","Imprecision","Heterogeneity","Incoherence"]
LZ:LZ+MET	1	Some concerns	Low risk	No concerns	Major concerns	No concerns	No concerns	Very low	["Within-study bias","Imprecision"]
MET:PIO	1	Some concerns	Low risk	No concerns	Major concerns	No concerns	Some concerns	Very low	["Within-study bias","Imprecision","Incoherence"]
CC:CC+MET+PIO	0	Some concerns	Low risk	No concerns	Some concerns	Some concerns	Some concerns	Very low	["Within-study bias","Imprecision","Heterogeneity","Incoherence"]
CC:CC+ROS	0	No concerns	Low risk	No concerns	Major concerns	No concerns	Some concerns	Very low	["Imprecision","Incoherence"]
CC:PIO	0	Some concerns	Low risk	No concerns	Major concerns	No concerns	Some concerns	Very low	["Within-study bias","Imprecision","Incoherence"]
CC+MET:PIO	0	Some concerns	Low risk	No concerns	Major concerns	No concerns	Some concerns	Very low	["Within-study bias","Imprecision","Incoherence"]

CC+MET+PIO: CC+ROS	0	Some concerns	Low risk	No concerns	Major concerns	No concerns	Some concerns	Very low	["Within-study bias","Imprecision","Incoherence"]
CC+MET+PIO: LZ	0	Some concerns	Low risk	No concerns	Major concerns	No concerns	Some concerns	Very low	["Within-study bias","Imprecision","Incoherence"]
CC+MET+PIO: LZ+MET	0	Some concerns	Low risk	No concerns	Major concerns	No concerns	Some concerns	Very low	["Within-study bias","Imprecision","Incoherence"]
CC+MET+PIO: MET	0	Some concerns	Low risk	No concerns	No concerns	Major concerns	Some concerns	Very low	["Within-study bias","Heterogeneity","Incoherence"]
CC+MET+PIO:PIO	0	Some concerns	Low risk	No concerns	Major concerns	No concerns	Some concerns	Very low	["Within-study bias","Imprecision","Incoherence"]
CC+ROS:LZ	0	No concerns	Low risk	No concerns	Major concerns	No concerns	Some concerns	Very low	["Imprecision","Incoherence"]
CC+ROS:LZ+MET	0	Some concerns	Low risk	No concerns	Major concerns	No concerns	Some concerns	Very low	["Within-study bias","Imprecision","Incoherence"]

CC+ROS:MET	0	No concerns	Low risk	No concerns	Major concerns	No concerns	Some concerns	Very low	["Imprecision","Incoherence"]
CC+ROS:PIO	0	Some concerns	Low risk	No concerns	Major concerns	No concerns	Some concerns	Very low	["Within-study bias","Imprecision","Incoherence"]
LZ:MET	0	Some concerns	Low risk	No concerns	Major concerns	No concerns	Some concerns	Very low	["Within-study bias","Imprecision","Incoherence"]
LZ:PIO	0	Some concerns	Low risk	No concerns	Major concerns	No concerns	Some concerns	Very low	["Within-study bias","Imprecision","Incoherence"]
LZ+MET:MET	0	Some concerns	Low risk	No concerns	Major concerns	No concerns	Some concerns	Very low	["Within-study bias","Imprecision","Incoherence"]
LZ+MET:PIO	0	Some concerns	Low risk	No concerns	Major concerns	No concerns	Some concerns	Very low	["Within-study bias","Imprecision","Incoherence"]

C. Grade of miscarriage

Comparison	Number of studies	Within-study bias	Reporting bias	Indirectness	Imprecision	Heterogeneity	Incoherence	Confidence rating	Reason(s) for downgrading
MET:Placebo	2	Some concerns	Low risk	No concerns	Major concerns	No concerns	No concerns	Very low	["Within-study bias","Imprecision"]
CC:Placebo	0	Some concerns	Low risk	No concerns	Major concerns	No concerns	No concerns	Very low	["Within-study bias","Imprecision"]
CC+MET:Placebo	0	Some concerns	Low risk	No concerns	Major concerns	No concerns	No concerns	Very low	["Within-study bias","Imprecision"]
CC+MET+PIO:Placebo	0	Some concerns	Low risk	No concerns	Major concerns	No concerns	No concerns	Very low	["Within-study bias","Imprecision"]
CC+ROS:Placebo	0	No concerns	Low risk	No concerns	Major concerns	No concerns	No concerns	Low	["Imprecision"]
LZ:Placebo	0	Some concerns	Low risk	No concerns	Major concerns	No concerns	No concerns	Very low	["Within-study bias","Imprecision"]
LZ+MET:Placebo	0	Some concerns	Low risk	No concerns	Major concerns	No concerns	No concerns	Very low	["Within-study bias","Imprecision"]
PIO:Placebo	0	Some concerns	Low risk	No concerns	Major concerns	No concerns	No concerns	Very low	["Within-study bias","Imprecisi

									on"]
CC:CC+MET	7	Some concerns	Low risk	No concerns	Some concerns	No concerns	No concerns	Low	["Within-study bias","Imprecision"]
CC:LZ	4	No concerns	Low risk	No concerns	Some concerns	No concerns	No concerns	Moderate	["Imprecision"]
CC:LZ+MET	1	Some concerns	Low risk	No concerns	Some concerns	No concerns	No concerns	Low	["Within-study bias","Imprecision"]
CC:MET	5	No concerns	Low risk	No concerns	Major concerns	No concerns	No concerns	Low	["Imprecision"]
CC+MET:CC+MET+PIO	1	Some concerns	Low risk	No concerns	Major concerns	No concerns	No concerns	Very low	["Within-study bias","Imprecision"]
CC+MET:CC+ROS	1	No concerns	Low risk	No concerns	Major concerns	No concerns	No concerns	Low	["Imprecision"]
CC+MET:LZ	1	Some concerns	Low risk	No concerns	Major concerns	No concerns	No concerns	Very low	["Within-study bias","Imprecision"]
CC+MET:LZ+MET	1	Some concerns	Low risk	No concerns	Major concerns	No concerns	No concerns	Very low	["Within-study bias","Imprecision"]
CC+MET:MET	4	No concerns	Low risk	No concerns	Some concerns	Some concerns	No concerns	Low	["Imprecision","Heterogeneity"]
LZ:LZ+MET	1	Some concerns	Low risk	No concerns	Major concerns	No concerns	No concerns	Very low	["Within-study bias","Imprecisi

									on"]
MET:PIO	1	Some concerns	Low risk	No concerns	Major concerns	No concerns	No concerns	Very low	["Within-study bias","Imprecision"]
CC:CC+MET+PIO	0	Some concerns	Low risk	No concerns	Major concerns	No concerns	No concerns	Very low	["Within-study bias","Imprecision"]
CC:CC+ROS	0	No concerns	Low risk	No concerns	Major concerns	No concerns	No concerns	Low	["Imprecision"]
CC:PIO	0	Some concerns	Low risk	No concerns	Major concerns	No concerns	No concerns	Very low	["Within-study bias","Imprecision"]
CC+MET:PIO	0	Some concerns	Low risk	No concerns	Major concerns	No concerns	No concerns	Very low	["Within-study bias","Imprecision"]
CC+MET+PIO: CC+ROS	0	Some concerns	Low risk	No concerns	Major concerns	No concerns	No concerns	Very low	["Within-study bias","Imprecision"]
CC+MET+PIO: LZ	0	Some concerns	Low risk	No concerns	Major concerns	No concerns	No concerns	Very low	["Within-study bias","Imprecision"]
CC+MET+PIO: LZ+MET	0	Some concerns	Low risk	No concerns	Major concerns	No concerns	No concerns	Very low	["Within-study bias","Imprecision"]
CC+MET+PIO: MET	0	Some concerns	Low risk	No concerns	Major concerns	No concerns	No concerns	Very low	["Within-study bias","Imprecisi

									on"]
CC+MET+PIO:PIO	0	Some concerns	Low risk	No concerns	Major concerns	No concerns	No concerns	Very low	["Within-study bias","Imprecision"]
CC+ROS:LZ	0	No concerns	Low risk	No concerns	Major concerns	No concerns	No concerns	Low	["Imprecision"]
CC+ROS:LZ+MET	0	Some concerns	Low risk	No concerns	Major concerns	No concerns	No concerns	Very low	["Within-study bias","Imprecision"]
CC+ROS:MET	0	No concerns	Low risk	No concerns	Major concerns	No concerns	No concerns	Low	["Imprecision"]
CC+ROS:PIO	0	No concerns	Low risk	No concerns	Major concerns	No concerns	No concerns	Low	["Imprecision"]
LZ:MET	0	No concerns	Low risk	No concerns	Major concerns	No concerns	No concerns	Low	["Imprecision"]
LZ:PIO	0	Some concerns	Low risk	No concerns	Major concerns	No concerns	No concerns	Very low	["Within-study bias","Imprecision"]
LZ+MET:MET	0	Some concerns	Low risk	No concerns	Some concerns	Some concerns	No concerns	Very low	["Within-study bias","Imprecision","Heterogeneity"]
LZ+MET:PIO	0	Some concerns	Low risk	No concerns	Major concerns	No concerns	No concerns	Very low	["Within-study bias","Imprecision"]

D. Grade of Ectopic pregnancy

Comparison	Number of studies	Within-study bias	Reporting bias	Indirectness	Imprecision	Heterogeneity	Incoherence	Confidence rating	Reason(s) for downgrading
MET:Placebo	1	No concerns	Low risk	No concerns	Major concerns	No concerns	No concerns	Low	["Imprecision"]
CC:Placebo	0	No concerns	Low risk	No concerns	Major concerns	No concerns	No concerns	Low	["Imprecision"]
CC+MET:Placebo	0	No concerns	Low risk	No concerns	Major concerns	No concerns	No concerns	Low	["Imprecision"]
LZ:Placebo	0	No concerns	Low risk	No concerns	Major concerns	No concerns	No concerns	Low	["Imprecision"]
LZ+MET:Placebo	0	Some concerns	Low risk	No concerns	Major concerns	No concerns	No concerns	Very low	["Within-study bias","Imprecision"]
CC:CC+MET	5	Some concerns	Low risk	No concerns	Major concerns	No concerns	No concerns	Very low	["Within-study bias","Imprecision"]
CC:LZ	4	No concerns	Low risk	No concerns	Major concerns	No concerns	No concerns	Low	["Imprecision"]
CC:LZ+MET	1	Some concerns	Low risk	No concerns	Major concerns	No concerns	No concerns	Very low	["Within-study bias","Imprecision"]

CC:MET	4	Some concerns	Low risk	No concerns	Major concerns	No concerns	No concerns	Very low	["Within-study bias","Imprecision"]
CC+MET:LZ	1	Some concerns	Low risk	No concerns	Major concerns	No concerns	No concerns	Very low	["Within-study bias","Imprecision"]
CC+MET:LZ+MET	1	Some concerns	Low risk	No concerns	Major concerns	No concerns	No concerns	Very low	["Within-study bias","Imprecision"]
CC+MET:MET	4	Some concerns	Low risk	No concerns	Major concerns	No concerns	No concerns	Very low	["Within-study bias","Imprecision"]
LZ:LZ+MET	1	Some concerns	Low risk	No concerns	Major concerns	No concerns	No concerns	Very low	["Within-study bias","Imprecision"]
LZ:MET	0	Some concerns	Low risk	No concerns	Major concerns	No concerns	No concerns	Very low	["Within-study bias","Imprecision"]
LZ+MET:MET	0	Some concerns	Low risk	No concerns	Major concerns	No concerns	No concerns	Very low	["Within-study bias","Imprecision"]

E. Grade of Multiple pregnancy

Comparison	Number of studies	Within-study bias	Reporting bias	Indirectness	Imprecision	Heterogeneity	Incoherence	Confidence rating	Reason(s) for downgrading
MET:Placebo	1	No concerns	Low risk	No concerns	Major concerns	No concerns	No concerns	Low	["Imprecision"]
CC:Placebo	0	No concerns	Low risk	No concerns	Major concerns	No concerns	No concerns	Low	["Imprecision"]
CC+MET:Placebo	0	No concerns	Low risk	No concerns	Major concerns	No concerns	No concerns	Low	["Imprecision"]
CC+MET+PIO:Placebo	0	Some concerns	Low risk	No concerns	Major concerns	No concerns	No concerns	Very low	["Within-study bias","Imprecision"]
CC+ROS:Placebo	0	No concerns	Low risk	No concerns	Major concerns	No concerns	No concerns	Low	["Imprecision"]
LZ:Placebo	0	No concerns	Low risk	No concerns	Major concerns	No concerns	No concerns	Low	["Imprecision"]
CC:CC+MET	6	Some concerns	Low risk	No concerns	Major concerns	No concerns	No concerns	Very low	["Within-study bias","Imprecision"]
CC:LZ	3	No concerns	Low risk	No concerns	Major concerns	No concerns	No concerns	Low	["Imprecision"]
CC:MET	3	Some concerns	Low risk	No concerns	Major concerns	No concerns	No concerns	Very low	["Within-study bias","Imprecision"]

CC+MET:CC+MET+PIO	1	Some concerns	Low risk	No concerns	Major concerns	No concerns	No concerns	Very low	["Within-study bias","Imprecision"]
CC+MET:CC+ROS	1	No concerns	Low risk	No concerns	Major concerns	No concerns	No concerns	Low	["Imprecision"]
CC+MET:MET	3	Some concerns	Low risk	No concerns	Major concerns	No concerns	No concerns	Very low	["Within-study bias","Imprecision"]
CC:CC+MET+PIO	0	Some concerns	Low risk	No concerns	Major concerns	No concerns	No concerns	Very low	["Within-study bias","Imprecision"]
CC:CC+ROS	0	No concerns	Low risk	No concerns	Major concerns	No concerns	No concerns	Low	["Imprecision"]
CC+MET:LZ	0	No concerns	Low risk	No concerns	Major concerns	No concerns	No concerns	Low	["Imprecision"]
CC+MET+PIO: CC+ROS	0	Some concerns	Low risk	No concerns	Major concerns	No concerns	No concerns	Very low	["Within-study bias","Imprecision"]
CC+MET+PIO: LZ	0	Some concerns	Low risk	No concerns	Major concerns	No concerns	No concerns	Very low	["Within-study bias","Imprecision"]
CC+MET+PIO: MET	0	Some concerns	Low risk	No concerns	Major concerns	No concerns	No concerns	Very low	["Within-study bias","Imprecision"]
CC+ROS:LZ	0	No concerns	Low risk	No concerns	Major concerns	No concerns	No concerns	Low	["Imprecision"]

CC+ROS:MET	0	No concerns	Low risk	No concerns	Major concerns	No concerns	No concerns	Low	["Imprecision"]
LZ:MET	0	No concerns	Low risk	No concerns	Major concerns	No concerns	No concerns	Low	["Imprecision"]
